# COVID-19 Pandemic Impact on Substance Misuse: A Social Media Listening, Mixed Method Analysis

**DOI:** 10.3390/brainsci11070907

**Published:** 2021-07-09

**Authors:** Davide Arillotta, Amira Guirguis, John Martin Corkery, Norbert Scherbaum, Fabrizio Schifano

**Affiliations:** 1 Psychopharmacology, Drug Misuse, and Novel Psychoactive Substances Research Unit, School of Life and Medical Sciences, University of Hertfordshire, Hatfield AL10 9AB, UK; davide.arillotta@yahoo.it (D.A.); a.guirguis2@herts.ac.uk (A.G.); j.corkery@herts.ac.uk (J.M.C.); F.Schifano@herts.ac.uk (F.S.); 2 Swansea University Medical School, Institute of Life Sciences 2, Swansea University, Singleton Park, Swansea SA2 8PP, UK; 3 Department of Psychiatry and Psychotherapy, Medical Faculty, LVR-Hospital Essen, University of Duisburg-Essen, Virchowstraße 174, 45147 Essen, Germany

**Keywords:** COVID-19, drug misuse, mixed methods, NPS, psychonauts, reddit

## Abstract

The restrictive measures adopted during the COVID-19 pandemic modified some previously consolidated drug use patterns. A focus on social networks allowed drug users to discuss, share opinions and provide advice during a worldwide emergency context. In order to explore COVID-19-related implications on drug trends/behaviour and on most popular psychotropic substances debated, the focus here was on the constantly updated, very popular, Reddit social platform’s posts and comments. A quantitative and qualitative analysis of r/Drugs and related subreddits, using a social media listening netnographic approach, was carried out. The post/comments analysed covered the time-frame December 2019–May 2020. Between December 2019 and May 2020, the number of whole r/Drugs subreddit members increased from 619,563 to 676,581 members, respectively, thus increasing by 9.2% by the end of the data collection. Both the top-level r/Drugs subreddit and 92 related subreddits were quantitatively analysed, with posts/comments related to 12 drug categories. The drugs most frequently commented on included cannabinoids, psychedelics, opiates/opioids, alcohol, stimulants and prescribed medications. The qualitative analysis was carried out focussing on four subreddits, relating to some 1685 posts and 3263 comments. Four main themes of discussion (e.g., lockdown-associated immunity and drug intake issues; drug-related behaviour/after-quarantine plans’ issues; lockdown-related psychopathological issues; and peer-to-peer advice at the time of COVID-19) and four categories of Redditors (e.g., those continuing the use of drugs despite the pandemic; the “couch epidemiologists”; the conspirationists/pseudo-science influencers; and the recovery-focused users) were tentatively identified here. A mixed-methods, social network-based analysis provided a range of valuable information on Redditors’ drug use/behaviour during the first phase of the COVID-19 pandemic. Further studies should be carried out focusing on other social networks as well as later phases of the pandemic.

## 1. Introduction

Over the last 16 months, the SARS-CoV-2 contagion has spread worldwide, with a number of measures (e.g., social distancing and quarantine; [1]) having been taken as a result. In association with self-confinement at home, many users have increased their web access on a daily basis [2,3]. Users have turned to the internet for news, exchange of views and purchase of products, with increased levels of the associated risk of exposure to unverified sources of information [4,5,6,7]. Within the open web, in addition to common search engines, there are social networks/platforms. These include Twitter, Facebook, Telegram, Youtube, WhatsApp and Reddit [8,9,10,11,12,13].

Reddit is one of the most used English-language social media/platforms; in May 2020 it counted some 430 million active users and over 1.5 billion visits. Its usage is more prevalent among young (18–29 years old), white and Hispanic males, typically with some level of high school/college education. Most Redditors (Reddit users) are located in the United States (49.8%), followed by the United Kingdom and Canada (respectively at 8.2 and 7.6%), Australia (3.7%) and Germany (3.1%; [14,15,16,17,18,19,20]). Although the Reddit platform does not necessarily represent either the general, or the whole drug enthusiasts’ population, it still appears as a large open web information source in terms of the number of posts and active users; variety of topics being discussed; and real-time discussion of trends. Hence, Reddit has been used for a range of research purposes, including both general psychiatry [21,22,23,24,25,26,27,28] and addiction/drug abuse [29,30,31,32,33,34]. Indeed, the social networks’ web monitoring approach has been suggested to be helpful to better understand some clinical and psychopharmacological issues related to a range of novel psychoactive substances (NPS) [35]. This approach has been recently referred to as “social media listening” [36,37], with the resulting findings providing insight into how people discuss their condition online. This research approach is, however, being combined with qualitative research for added rigour and confirmation of relevance of the related findings, i.e., triangulation [37]. Over the years, netnography, a qualitative, interpretative research methodology that adapts traditional ethnographic techniques to the study of online (e.g., social media; web) data [38] has increasingly been used as a strictly observational, unobtrusive approach in a range of health research fields, including pro-anorexia [39] and diabetes mellitus online peer support [40] communities. Overall, web monitoring activities may well help in identifying a wide range of emerging/novel/previously undetected NPS [41,42,43,44]. A particular type of active user identified from the various virtual platforms are the e-psychonauts [45]. Their interest is addressed to the entire world of misusing substances, ranging from the classic ones to emerging psychoactive substances (EPS) [45,46,47,48]. The knowledge levels relating to the ever-growing number of NPS/EPS, motivations for use, sought-after psychoactive effects, idiosyncratic combinations, medical/psychopathological risks and clinical treatment/management are limited [48,49,50,51,52,53]. It has been suggested [54] that the current pandemic is having an impact on the supply of substances of abuse and their consumption, with the overall social, economic and health consequences of the pandemic itself not yet foreseeable [55,56,57].

### Aims

We aimed here at providing an overview of both the content/quality and quantity of posts/comments relating to the psychotropic substances debate detectable from the Reddit platform during the early phase of the SARS-Cov-2 pandemic.

## 2. Materials and Methods

A quantitative and qualitative analysis of Reddit subreddits was conducted from December 2019 to May 2020. In order to investigate drug use issues among e-psychonauts, other Reddit users and the related possible COVID-19 pandemic impact, a mixed-methods (MM) strategy was adopted. With this approach, a range of qualitative and quantitative data are analysed in parallel to provide a better understanding of a specific issue [58,59,60]. More specifically, the quantitative component was here the result of the manual screening of Reddit posts/threads, whilst for the qualitative analysis a netnographic [38,39,40,41,42,43,44,45,46,47,48,49,50,51,52,53,54,55,56,57,58,59,60,61] approach was adopted. In this study, original posts and comments were included. In Reddit, “post” means the first content shared by a user (e.g., questions, thoughts, tips, experiences, pictures) and “comment” defines any eventual reply to this specific post. Data were collected from both the Reddit website and the r/Drugs’ related subreddits; conversely, the r/covid19stack and r/Opioid_RCs subreddits were located from dedicated links [62,63,64,65,66,67]. More precisely, D.A. carried out the initial posts/comments’ extraction activity; the range of free texts identified were screened and their proper, initial coding/categorization was drafted by D.A., a psychiatrist with quantitative/qualitative research experience assessing web-based NPS drug discussions. The findings and categorizations were then independently reviewed by F.S., a senior academic with proper teaching and research experience on quantitative/qualitative recreational/NPS drug research, in order to achieve an agreement to be presented to the whole researchers’ group for both resolving possible disagreements and final approval of the quantitative analysis.

The quantitative search, relating to the r/Drugs Reddit component and its related subreddits [66], was performed by inserting specific keywords into the search bar of each subreddit. This process included two phases. In the first phase, the latest/trendiest topics were explored, in order to obtain the keywords that were eventually used to refine further searches. The keywords first used here included “COVID”, “coronavirus”, “corona”, “quarantine”, “pandemic” and “lockdown”. In the second phase, the titles/contents of the discussions were explored, whilst analysing the presence of the above mentioned COVID-19-related keywords in all the r/Drugs subreddits. The threads adopting the “New” posts order criteria were screened here to facilitate optimal temporal discrimination. The Reddit new/trendy topics can be explored by considering the ranking functions (e.g., Hot, New, Top, Rising). This option allowed the exclusion of those posts/comments that were clearly antecedent to the beginning of the pandemic. Irrelevant/not applicable posts (e.g., where the term “*corona*” referred to a brand of an alcoholic drink) were excluded. To facilitate the manual keyword search, an Application Programming Interface (API), developed by Reddit users and hosted in a distinct website [68] (GitHub, 2020), was used. The search was repeated several times, a thousand to a thousand items at a time, and, hence, the 1000 item limit imposed by the native Reddit API [67] was bypassed (see Table S1).

### 2.1. Qualitative Analysis

The qualitative component of the research was carried out employing the netnographic approach. The qualitative analysis of both the whole r/Drugs subreddit and some related, most popular subreddits (r/HPPD; r/StackAdvice; r/Supplements) was conducted (see details in Table 1 and Table S2). The themes of these subreddits revolved around (a) general issues related to the range of available psychotropics; (b) the specific psychopathological consequences of drug intake; (c) the range of remedies considered by Reddit users in coping with untoward consequences of drug intake; and (d) peer-to-peer advice and social media sharing issues. Free texts (e.g., posts/comments) containing the selected keywords were examined; some representative quotes were then identified and grouped in the “main themes” Results section. A similar procedure was carried out to identify and group the different “types of users”. In line with previous suggestions [69,70], the posts were selected whilst applying different and multiple sampling strategies. More precisely, the Palinkas et al. [7] theoretical sampling criteria were considered here to group the different types of users, whilst multistage (e.g., opportunistic or emergent) sampling criteria were considered to identify the main themes. The iterative process made it possible to refine and classify the posts/comments as representative for putative categories, until their screening was completed. The groups’ classification was carried out taking into account the contents’ variety of COVID-19 keyword-related posts using an inductive approach (e.g., generation of new theory emerging from the data; [71,72]).

### 2.2. Data Collection and Privacy Disclaimer

The current overall screening focussed on Redditors’ COVID-19-related activities running from 01 December 2019 to 31 May 2020, in order to include the first six months of the COVID-19 outbreak [73]. The precise number of members made available by Reddit related only to the date in which the social platform was being accessed; conversely, no historical data of members’ figures were made available from the Reddit platform. Hence, to better assess the possible levels of numerical fluctuations relating to both selected subreddits’ members and posts/comments, the quantitative analysis was carried on the 11th day of each month, using 11th March 2020 as a time-point reference, with this date having been the date on which the WHO declared COVID-19 to be a pandemic ([74]; see Table S1).

Each subreddit content was freely accessible on the open web. In line with previous studies [27], full anonymity was guaranteed and no usernames or references to the Redditors were collected, used or analysed. The raw data were imported into Word and Microsoft Excel spreadsheets. Furthermore, according to the unobtrusive and naturalistic methods of conducting netnographic studies [48,49,50,51,52,53,54,55,56,57,58,59,60,61], no posts or other contributions to private/public forum discussions were made.

### 2.3. Ethics

The study was carried out within the framework of the University of Hertfordshire Ethics’ Committee (code: aLMS/SF/UH/02951(2) approval).

## 3. Results

### 3.1. Number of Subreddits and Their Classification

On 11 December 2019 and 11 May 2020, 619,563 and 676,581 members were counted on the whole r/Drugs subreddit, respectively, with a 9.2% overall increase overtime.

### 3.2. Included and Excluded Subreddits

Some 217 subreddits related to r/Drugs [66] were identified. However, some 127 subreddits, including Pics & Videos, Music, Youtube, Festivals, Misc, Regions/Languages, Memes, 18+ & NSFW were excluded from the analysis because they were deemed not relevant. Most typically, in fact, these subreddits focussed on a range of issues including pornography, sex-mate searches, pictures, videos, memes and music festivals. Quantitative data regarding the remaining 90 subreddits were collected. Two further subreddits, albeit not explicitly listed within the macro r/Drugs subreddit itself but considered here as relevant (r/Opioid_RCs; r/covid19stack), were added, taking the total to 92 subreddits being analysed.

### 3.3. Clinical and Pharmacological Subreddits′ Classification

Given the large variety of drug issues being discussed in these subreddits, and in line with previous clinical pharmacological classification of recreational drugs/NPS/EPS [75], the above subreddits were grouped into 12 categories (see Table S3). Moreover, it was identified that 20 subreddits focussed on “psychedelics” (PS); 13 on “cannabinoids (CA)”; 10 on “GABAergics, gabapentinoids and various psychotropic medications (GM)”; 9 on “cognitive enhancers/image- and performance-enhancing drugs (IPEDs)/supplements (CS)”; 9 on “opioids (OP)”; 5 on “generic subreddits and drug-induced disorders (GD)”; 5 on “dissociatives (DI)”; 5 on “beverages/brews and infusions (BI)”; 5 on “alcohol and cigarettes (AC)”; 4 on “stimulants (ST)”; 3 on “research chemicals (RC)”; 2 on “entactogens (EN)”; and 2 on “other substances (OS)” subreddits. Within the “Addiction/Recovery”, “Harm Reduction” and “Discussion” subreddits, those with less than 100,000 entries were excluded from the quantitative analysis (see Table S4) because they were considered anecdotal/less representative.

Those drug categories that, in terms of posts’ numbers, received more attention included the following: CA, PS, OP, AC, ST; and, in terms of comments, the categories included the following: CA, OP, CS, PS, GM. Overall, the largest numbers of posts/comments were identified from the “Drugs & Activities” group.

Among the “Drugs A-Z” group, the largest number (n = 1,553,229) of Redditors related here to *r/trees*. Considering an arbitrary cut-off of 100 K members, in terms of popularity it was followed in decreasing order by r/LSD, r/Nootropics, r/shrooms, r/DMT and r/Supplements. Within the “Discussion” group, the largest number of members belonged to r/Psychonaut (n = 282,864), followed, with about/more than 100 K members, by r/vaporents and r/DrugNerds. Within the above-mentioned timeframe, the largest members’ number (e.g., in excess of 100%) increase was observed in the following subreddits: r/covid19stack, r/Opioid_RCs, r/mescaline and r/MephHeads.

### 3.4. Supplements and Remedies

Some 87 supplements (e.g., vitamins, minerals, herb/botanical/plant extracts, amino-acids, traditional medicine products) and 7 remaining non-chemical remedies were identified as modalities to boost the immune system (See Table 2). The supplements’ and remedies’ list was extracted from three subreddits included in the “Therapeutic use” section of “cognitive enhancers/PIEDS” group (r/covid19stack, r/StackAdvice and r/Supplements) (See Table S3).

According to the current keyword criteria search, the first COVID-19-related posts/comments were dated 22 January 2020 (r/steroids) and 23 January 2020 (r/ketamine; r/researchchemicals) (See Table S1); users commented about the related health/sanitizing concerns (e.g., (…) So a lot of street K is coming from China and I′d say its safe to say sanitation is probably not a criminal operation′s main priority (…) So, I wonder if its poasible to get contaiminated drugs from the area? (…)—Yeah I heard about the coronavirus. Scary shit! (…)).

For further details on members and individual keyword data on posts/comments, see Tables S1–S3.

### 3.5. Main Themes Being Discussed

Regarding cannabinoids, the focus was most typically on cultivation issues, whilst for psychedelics, cognitive enhancers and GABAergics/prescribed/over the counter (OTC) medications, the discussion revolved around the large range of related molecules. Conversely, NPS/EPS were typically listed in the “research chemicals” group, and, in particular, r/researchchemicals and r/Opioid_RCs. Finally, addiction recovery issues were identified from virtually all the analysed drug groups (for further details, see Table S1). Most typically, subredditors discussed a large range of themes, but four main themes were tentatively identified (see Table 2 and Table S2).

A total of four subreddits (relating to a total of 1685 posts and 3263 comments, as shown in Table 2) were screened for the qualitative analysis. From the whole list (see Table S2), they were selected according to the following criteria: r/Drugs, the main subreddit, since focussing on cross-cutting themes and general discussions; r/HPPD, the only r/Drugs related subreddit focussing on drug-induced disorders (Reddit, 2020 g); r/StackAdvice, dealing with the best drug stacking advice; r/Supplements, debating the different supplements and remedies to use to cope with possible drug/COVID-19-related untoward consequences. Due to the large volume of raw data generated, irrelevant posts/comments were excluded and manually removed from the data set. Following the removal of post/comments, the most relevant posts/comments (n = 190) were listed in Table S2: 69 on main theme one; 73 on main theme two; 16 on main theme three and 21 on main theme four. A brief summary of the issues debated within the four main themes is provided here (see also Table 3).

#### 3.5.1. Main Theme 1. The Lockdown-Associated: Immunity; Psychotropic(s) of Choice; and Drug Dosage/Intake Issues

Sub-theme 1. Drug use and impact on immunity levels. Table S2, page 1. The need to boost the immune response with specific drugs was a hot topic.

-  (…) Substances that boost immune response? (…) Im curious in both recreational and non recreational ones. (…) Edit: please provide evidence for claims while making them…….
-  (…) i read kratom actually boosts your immune system. I could swear thats why I never get sick lol—Maybe don′t do MDMA or other drugs that mess with your immune system);
-  (…) Hey, sorry is this is a dumb question, but: is there anything I can do to molly to make sure that COVID isn’t on it? Should I attempt to wash the pill, or?);
-  (…) A cocaine only diet provides all the nutrients you need. Food is just a burden for the weak).
-  (…) So cocaine doesn′t cure covid-19?; I doubt that vaping cannabis will affect your immune system very much.
-  (…) Anyone know if suboxone effects the immune system much? If the answer is yes don′t tell me, I get severe health anxiety. e.g., What if you have AD/HD and use amphetamines daily as treatment? Will this impact my immune system if I stick to the dosages I′ve been instructed to use by my physician?; e.g., Tripping with Schizophrenia (opinions?).
-  Sub-theme 2. Looking for the optimal recreational/prescribing drugs, alone or in combination, to cope with the pandemic. Table S2, pages 1–3.
-  (…) I am bored in quarantine and I am out of weed and benzo as my dealer got coronavirus and stopped delivering. I am looking for another way to get high—DMT, Shrooms, Ketamine, Edibles/Weed, Opioids, Benzos, Cocaine are all good indoor drugs.—Cant smoke in my house when parents are in, need a non smelly drug.
-  (…) What’s in your doomsday stash? What would you still like to add before lockdown happens (assuming we end up like Italy)? What kind of activities do you like to do while high to pass the time? Here’s a rundown of what I’ve got: Codeine 30 mg; lots of it. Weed in various forms from the dispensary (3 vape carts, 4 bags of gummies, 2 g of flower, 2 strains I grew myself, etc). CBD flower & isolate. Kratom; lots of it. Benzedrex inhalers × 2. Caffeine tablets, 200 mg. Xanax, 0.5 mg. Norco (as of yesterday, thanks dentist!), 7.5 mg × 20 tabs. Fioricet × about 20 tablets. Coca powder & tea bags × 100 g powder, 5 teabags. Various random drugs I don’t take (methocarbomol, alcohol, guanfacine, klonopin, phentermine).—I work from home right now, so cocaine is next to my workstation—Forced to detox off amphetamine because it doesnt get made in my country and every country in eu is on lockdown…Maybe i’ll turn into alcohol or weed for the time being who knows.)
-  (…) (e.g., a bit of weed + a sniff of midma + a bit of acid all while coming up on some that juicy rona fever 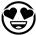

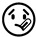
 stay safe but if you hit that rona make sure to stay hydrated 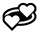 edit: ok i’m not funny this is just a joke about covid-19 fever: (; Phenibut + caffeine + weed + xanax is fucking awesome
-  (…)—Anybody else ever make “potions”? Examples would be ground klonopin in Kratom tea, maybe a splash of vodka or something. Wine, vodka, beer, vyvanse all mixed together. Etc, etc. Quarantine has clearly driven me crazy).

 Sub-theme 3. Drug dosage adjustment and intake modalities’ issues. Table S2, pages 3,4.

-  (…) Will i feel anything on 95 mg od codeine? … so i have A couple of codeine phosphas + sulfgogaiacolum shit will this get me high? I’m just so desperate cuz covid makes everything so hard to get—I can no longer workout without getting high.
-  (…) However due to the Covid-19 lockdown, I’ve had to workout at home. Being at home has led to me getting high on the reg, and before working out cause it feels AMAZING.
-  (…) I don’t feel the need to take the Tramadol generally, but to workout I feel like I need it).
-  (…) ADHD medication… Does anyone know if doctors are allowed to mail the prescription in the mail so I can take it to a pharmacy here?
-  (…) Quit Heroin due to quarantine. Don’t applaud, I’m still using all kinds of other shit.—Trams anyone? I have no access to my other vices except trams.
-  (…) Quitting Smoking (tobacco) During an Impossibly Stressful Time I’ve been trying to keep myself distracted, but I honestly wasn’t prepared for the intensity to which my depression has worsened as a result of this.—Nicotine withdrawals weren’t too bad).
-  (…) Too anxious to smoke up … Concentrates and herb vaping should be easier on your lungs just empty the joints if you have a vape. Also oral and sublingual methods, edibles/tincture/distillate.

#### 3.5.2. Main Theme 2. Life at the Time of COVID-19; Drug Dealing; Drug-Related Behaviour; and Post-Quarantine Plans’ Issues

Sub-theme 4. Drug supplying and “customer care” issues. Table S2, pages 4–5.

-  (…) My dealer just dropped off a whole care package for me, lsd, shrooms, shatter and dmt… The man knows what I like… Tells me he knows that times are tough, that being stuck at home sucks and he hopes this helps and didnt ask for a penny.
-  (…) They’ve gotten more expensive where I live—Where I live, supply and demand have both gone up. Shit’s popping off more than ever but they have no problems keeping it stocked
-  (…) I think the biggest impact will be on supply chain. Will dealers stop selling? Definitely not, that’d mean giving up a source of income in a time of high unemployment. The supply chain thus spreads the virus. Lockdowns and quarantines will not deter addicts.

 Sub-theme 5. Drug-related behavioural issues. Table S2, pages 5–8.

-  (…) I’m gonna use this quarantine to get sober and hopefully get back to just socially drinking/using—Please tell me I’m not the only one this desperate
-  (…) I scraped my grinder with a pocket knife for an hour and rolled a spliff that tasted like plastic. Due to my low tolerance it kinda worked though. These quarantine times got me doing desperate shit;).
-  (…) I finally wrote a report on DMT … I was stricken with worry about their fate amidst the COVID-19 pandemic. I began to project how I would feel if they were to pass from it, to empathize with that potential future trauma.—Worst time to trip
-  (…) Got crazy drunk last night, took a heroic dose of acid, throwing up on acid is rough, throwing up thinking you have corona virus is the absolute worse thing ever. Still tripping, life is definitely weird rn).
-  (…) I am not into conspiracies, but I can’t help thinking about e.g., DMT being illegal around the world.—Think about your National Health Service please
-  (…) You might think you know what you can handle but right now is not the time to risk it. This is for your benefit too—Nice haha, yeah I think this quarantine has affected everyone for better or for worse).

 Sub-theme 6. Post-quarantine plans. Table S2, page 8.

-  (…) How to Structure Multiple-Drug Trip (Weed, LSD, Shrooms, DMT, E)
-  (…) Me and a couple of friends are looking to go on an Airbnb trip post-quarantine (thinking of 5 days, not sure though) and we’re wondering how exactly we should structure our time).

#### 3.5.3. Main Theme 3. Lockdown-Related Psychopathological Issues

-  (…) a week after I got off the Methadone, the whole Covid-19 crap and other stuff in my life whacked me with hella stress, anxiety, and palpitations. Soooo I’m back on the dope:/just trying to not lose it completely. What I need is to find a psychiatrist and counselor—I have all the same things in my mind every day actually. I want to take some drugs but trying prevent me from this. Also i’m in mental struggle with myself that borders with the depression.
-  (…) HPPD keeps giving me a shit ton of anxiety, advice? (…) Now that i’m in quarantine i can’t stop thinking about it). Table S2, Pages 10–11

#### 3.5.4. Main Theme 4. Peer-to-Peer Advice at the Time of COVID-19

-  (…) Before you take a heroic dose or try a new drug/batch … Please make certain what you are doing is safe. Hospitals are more overloaded than usual due to COVID-19.
-  (…) Meditate for Peace, Health and Happiness. Develop Monk-Like Discipline and Peace of Mind During These Anxious and Uncertain Times. Wishing everyone all the love, joy and blessings
-  (…) Day #1—Join me in the Dopamine Fasting Challenge during the COVID Self Isolation!). Table S2, Pages 9–11

### 3.6. Categories/Types of Users

In analysing all of the posts and comments, four types of users were tentatively identified. Indeed, the COVID-19 pandemic seemed to have impacted the drug supply chain, drug choice and users’ behavioural changes in various ways. One could argue that there are differences in reacting to the pandemic due to individual characteristics, place of living and personality traits. Those topics most typically debated revolved around COVID-19 health-related concerns, reliable psychological/psychiatric indicators of distress, loneliness, the desire to obtain an altered state of mind to better cope with boredom and troubles in accessing proper medical prescriptions. Some examples are provided here, with further clinical vignettes available in Table 4.

#### 3.6.1. Type 1. Redditors Considering Continuing the Use of Drugs Despite the Pandemic

(…) Sorry, imma gonna keep doing drugs as usual.

#### 3.6.2. Type 2. Couch Epidemiologists

(…) I realized the other day that the early warning symptoms of coronavirus are basically identical to mild heroin withdrawals so… now I’ve just been kinda uncertain as to whether I actually have coronavirus or if (…) I just feel better when I hit the black cause it’s getting me well lmao yikes.

MDMA weakens your immun system. And I’m not sure how it is with amphetamine. The chance of dying by using mdma and getting Corona virus in 1–2 days after is bigger than 0.2%.

#### 3.6.3. Type 3. Conspirationists/Pseudo-Science Influencers

(…) The seed code is CORONA. Enjoy, psychonauts; Don′t worry, the CIA will continue to funnel in all yalls drugs……

(…) Can you ask the DMT entities to help us solve this COVID-19 situation?; Psychedelics are the cure.

#### 3.6.4. Type 4. Recovery-Focused Users

(…) Despite lockdown, and other posts on here, you DONT have to relapse. This time can be used for recovery instead…… e.g., Guys! Being sober can be really nice, too……

(…) Ok dad, I′ll stop the molly.

## 4. Discussion

The present research has been conducted on a major and very popular social network, covering a time-period of six months, aiming at exploring the relationship between COVID-19 and substance misuse through a social media listening approach. In order to outline a comprehensive picture of the phenomenon, a large number of posts/comments, using a netnographic approach, were analysed here. At present, although the pandemic has been going on for several months, the attention of both the scientific literature and the media has mostly been directed to its medical/virological aspects. Conversely, only a few efforts have been made to clarify the impact of the COVID-19-related lockdown on both mental health and patterns/styles of recreational drug intake. For this reason, the current research focus was on the first six months of the pandemic, months that have been characterized by the strongest levels of self-isolation. From this point of view, for some vulnerable individuals, the only possible travel at that time was a “trip”, whilst being connected with peers through social media.

With the very best of our understanding, the current paper is likely to represent the first mixed-methods, large sample size, empirical investigation focussing on behavioural changes in drug intake during the first six months of the COVID-19 pandemic using a social media listening approach. Other studies have aimed at describing the trends in substance use during the current pandemic [43,44,76,77,78,79,80,81], without considering the need to carry out large-scale, social media mining, research activities [82,83,84]. Per definition, lockdown has been, and for some countries still is, characterised by both social confinement and human isolation. In parallel, the use of both the web and social media has increased considerably to maintain human contacts, hence the importance of assessing the drug enthusiasts’ online entries [54]. As previously highlighted, the online and offline worlds influence each other [85,86], although the directionality and the extent of these phenomena are not always clear. For social listening, Reddit was chosen here as the platform to be investigated. Although the Reddit platform does not provide precise data relating to its total members during the period December 2019–May 2020, anecdotal data have suggested that Reddit, as a social platform, recorded a spike in engagement during the pandemic [87]. The interest in social media during the pandemic has been explored by recent remaining studies as well [88].

Different from other social networks such as Facebook, Twitter, etc., Reddit promotes anonymity, with some Redditors describing themselves in different fantasy ways (e.g., “friendly faces” in r/Drugs; “tryptonaut” in r/tryptonaut; “bartards” in r/benzodiazepines; “dextronauts” in r/dxm; “opiate enthusiasts” in r/opiates; “ents” in r/trees; “salvia people” in r/Salvia, “Vaporists” in r/vaporents, etc.). Indeed, these nicknames may emphasise the importance of the sense of belonging to a sub-culture, different from the remaining sub-tribes and, indeed, from the “druggies” as well.

Overall, Redditors showed a strong tendency to share with their peers a range of materials (e.g., pictures, links, trip reports), to facilitate mutual help activities (“sharing for caring, sharing for drugging”). Users′ posts and comments focussed on a range of COVID-19-related issues; health and sanitising issues were frequently mentioned, including the quality/possible contamination of drug products and the risks of health consequences [80,89] in those with underlying chronic medical conditions. Many users asked for some advice about the possible of use of specific supplements to boost their immune system [90,91,92,93,94] and, indeed, the current pandemic stimulated the search for a range of psychotropics with only limited levels of potential harm on both the respiratory and the immune systems. In line with this, more attention was given to oral, as opposed to inhaled, modes of intake. Furthermore, there were a large number of posts related to both the indoor cultivation (e.g., of cannabis) and intake of edible products (see Table 2).

The recent literature has highlighted the dramatic impact of COVID-19 on mental health [95,96,97,98]. In line with this, various posts related to a range of possible concerns (e.g., anxiety, depressive, psychotic, hypochondriac, obsessive-compulsive), although this was not necessarily associated with a modification of Redditors’ drug consumption patterns. A special focus of the debate was here on the Hallucinogen Persisting Perception Disorder (HPPD) [99,100], with users asking for remedies to treat the problem, due to the impossibility of visiting clinicians/specialists.

COVID-19 affected drug markets in various ways, with sudden changes in drug purity/potency, price and supply/availability levels [80,101,102]. These issues were hotly debated here, especially in some subreddit groups (e.g., r/Addiction; r/Recovery). Although no obvious drug exchange elements were reported here, likely because these activities are explicitly discouraged by Reddit moderators with a penalty consisting of being banned, characteristics of drug delivery activities were reported, with specific reference to some dealers’ optimal “customer care” approach.

Overall, the quarantine regime seemed to have encouraged Redditors to develop a range of consumption coping strategies, including conservative (e.g., continuing with drug intake using stocks of purchased drugs); a switch to NPS, which can be bought online and delivered by the postman whilst avoiding legal issues; abandoning drug use and looking for new alternative activities (e.g., studying, doing physical exercise/workout) to be put in place to cope with the lack of psychoactive drugs. Interestingly, whilst some users tried to minimise the risks related to the modified lifestyle scenario, others showed relative levels of carelessness, with several comments having been related here to a range of conspiracy theories, otherwise widely disseminated in social media [103,104].

Whilst all the drug categories attracted Redditors’ attention, cannabis, alcohol, psychedelics, opioids and cognitive enhancers were the most popular drugs [105]. Due to their availability levels, alcohol and cannabis were often cited as replacement, “comfort”, psychotropics. Conversely alcohol polydrug use [106], to obtain an intense dissociative/detachment from the current contingency, was noted as having increased during lockdown.

Although some users seemed to be cautious about using substances at home during the pandemic, for others (e.g., “psychonauts”; [43,44,47,107,108]), the confinement experience triggered the use of NPS and/or psychedelics to facilitate insight and mental exploration issues. Indeed, some r/DrugNerds and r/researchchemicals Redditors seemed to focus on a compulsive search for new molecules to be tried. The availability of such posts and comments may have contributed to both the spread of “infodemia” [109] and to the related risks associated with the intake of NPS presenting with only little, or none at all, clinical pharmacological knowledge [75,110,111,112].

Consistent with previous research [54,113], significant at home, lockdown-related levels of use of both prescribed and OTC sedative drugs during the COVID-19 pandemic were identified. One could speculate that this was carried out to obtain an ad hoc, cloudy/obtunded (“four walls’ daze”; Yan et al., 2020), state of mind to best cope with both boredom and loneliness. In other cases, the switch to the psychotropics available at home, such as the prescribed/OTC medications, was carried out because of the lack of access to the preferred recreational drugs(s). On the other hand, many users complained that they were unable to renew their prescriptions, for the impossibility of going outside/visiting a doctor.

Whilst pre-COVID-19, web-based drug enthusiasts’ categorisations had already been made available [81], an attempt at categorizing Redditors with respect to their approach to drug use at the time of COVID-19 is proposed. Although the importance of personality traits is, indeed, relevant in deriving categories of drug users, assessing the Redditors’ personality dimensions was not the aim of the current study [114,115,116,117,118,119]. Specific personality issues could well have influenced the single subjects’ drug use intake/related behaviour during the pandemic itself. Indeed, reference to specific temperament and character traits, including the four dimensions of temperament (e.g., novelty seeking, harm avoidance, reward dependence and persistence); and the three dimensions of character (e.g., self-directedness, cooperativeness and self-transcendence; [120,121,122,123,124,125,126]) could have been of help here in interpreting the range of different drug-related behaviour described. These behaviours included persisting in careless drug use, arguably suggesting low levels of harm avoidance; high novelty seeking levels, possibly characterising those attracted by NPS; high self-transcendence levels in those looking for NPS/novel psychedelics; antisocial personality traits in those allegedly refusing to wear a face mask whilst in others’ presence [127]; with neuroticism, hypochondria and “coronaphobia” in those in need to be reassured whilst still using drugs [128,129,130,131,132]. Furthermore, the different attitudes described here could have been associated as well with the subject’s environmental/social context, family/work background, age [48], attachment styles and cognitive biases [116,133,134].

### Limitations

The current study presented with a few limitations. First, the current approach was adopted whilst considering both the empirical phenomenology model [135] and grounded theory principles [136,137], although one could argue the investigation was not fully in line with these approaches. Furthermore, the quantitative/qualitative posts/comments’ assessment was carried out manually, whilst ad hoc software to analyse specific subreddits was used before [34,138,139,140]. Conversely, the manual searching approach likely enhanced the value of the entries’ qualitative assessment here, fully in line with netnographic analysis principles [35,141]. On social media, people tend to speak freely and that by itself is an advantage, unlike other methods of obtaining data such as focus groups or interviews. On the other side, social data analysis may imply biases, methodological pitfalls and ethic boundaries [142]; for, e.g., it could be argued that Redditors represent an extremely varied population, potentially including a large plethora of users such as e-psychonauts, academics/researchers, curious people, trolls and trend influencers. Moreover, social media/Reddit users are only partially representative of people who use drugs in general; for, e.g., as recently highlighted, not all persons with drug-related problems have access to PCs/smartphones [143]. Finally, only the first few months of the COVID-19 pandemic were analysed here, and, at the time of writing, this pandemic is not over yet.

## 5. Conclusions

The current mixed-methods, qualitative and quantitative, focus on Reddit entries provided a range of valuable information relating to drug use at the time of COVID-19, thus confirming that social media analysis allows both trend and real-time data to be obtained. This approach is potentially useful for a wide range of researchers, clinicians and stakeholders, e.g., psychiatrists, psychologists, pharmacologists, sociologists, harm reduction projects. A tentative Redditors’ categorisation has also been proposed. Personality traits/cognitive bias, attachment styles, health/psychiatric worries, medical access issues, living/consumption settings and pressure to expose contents/thoughts could probably explain the different detected reactions. The Reddit community seems to auto-regulate itself with harm reduction advice, but various drug users’ trending patterns could worry clinicians and institutions. Further studies should be carried out by adding country-specific keywords, more subreddits/drug themes and focusing on other social networks as well. More studies should expand the potential of this approach in systematically exploring social networks in order to offer ideas for further investigations. Understanding the cultural, behavioural and psychological nuances/patterns related to the multi-faceted world of drug consumption, could help in designing new drug treatment/management and prevention strategies tailored to peculiar/new/changing contexts such as the COVID-19 pandemic.

## Figures and Tables

**Table 1:** Included subreddits in qualitative analysis.

Subreddits	COVID Posts/Comments	Coronavirus Posts/Comments	Corona Posts/Comments	Quarantine Posts/Comments	Pandemic Posts/Comments	Lockdown Posts/Comments	Total Posts/Comments
**r/Drugs**	158/538	227/449	210/402	556/645	127/195	148/239	1426/2468
**r/HPPD**	8/7	5/16	6/9	11/15	6/4	3/14	39/65
**r/StackAdvice**	16/31	6/10	3/10	2/12	4/12	1/5	32/80
**r/Supplements**	81/323	54/187	17/61	16/32	16/37	4/10	188/650
**Posts/comments**	263/899	292/662	236/482	585/704	153/248	156/268	1685/3263

**Table 2:** Supplements and remedies in alphabetical order discussed in r/covid19stack, r/Supplements and r/StackAdvice.

**A.** **Vitamins, Plants and Supplements**
1. Neuro magnesium
2. Apple cider vinegar
3. Artemisia
4. Ascorbyl palmitate
5. Ashwagandha
6. Astaxanthin (krill oil)
7. Astralgus
8. Baicalin
9. Beta 1,3 d glucan
10. Bismuth complexes
11. Black elderberry
12. Black seed
13. Black tea
14. Boron
15. Cat’s claw
16. Chaga
17. Chinese herb stack of mostly huangqi, gancao, fangfeng, baizhu, jinyinhua and lianqiao)/supportive medicines widely used in China (blend of ephedra, ginger, liquorice, various mushrooms)
18. Chlorella
19. Chocolate
20. Citicoline
21. Clinoptilolite
22. Colloidal silver
23. Colostrum
24. Cordyceps
25. Creatine
26. Curcumin
27. D, l-lysine acetylsalicylate and glycine
28. Echinacea
29. Elderberry juice/syrup
30. Elderberry/black elderberry
31. Epicor
32. Essential oils
33. Exogenous ketones
34. Five defenders
35. Food—originating ace inhibitors, including antihypertensive peptides, as preventive food components/foods to boost nitric oxide levels
36. Gaba
37. Garlic (allium sativum)
38. Green tea catechins/egcg
39. Herbal treatments
40. Honey
41. Iodine (lugol′s)
42. Iota-carrageenan nasal spray
43. Iron
44. K2
45. Khavinson peptides such as honoluten
46. Kimchee
47. Ldn
48. Liquorice root/liquorice root extract (or just glycyrrhizin)
49. L-tyrosine
50. Luteolin
51. Main protease (mpro) inhibitors from natural polyphenols
52. Manuka
53. Melatonin
54. Methylene blue
55. Monolaurin
56. Mormon tea
57. Nac (n-acetylcysteine)
58. Nigella sativa
59. Nitric oxide/naturally produced nitric oxide
60. Omega 3
61. Palmitoylethanolamide
62. Pelargonium juice/pelargonium sidoides
63. Povidone iodine
64. Probiotics
65. Ps-100
66. Quercetin
67. Rainbow light counterattack
68. Reishi mushrooms
69. Resveratrol
70. Sambucus nigra extracts
71. Selenium
72. Senolytics in general dasatinib/querecetin or foxo4-dri
73. Skullcap
74. Spirulina
75. Sulforaphane
76. Taurine.
77. Thymosin alpha 1
78. Tinospora cordifolia
79. Turkey tail
80. Turmeric
81. Turpentine
82. Vitamin a
83. Vitamin b complex/b 12/b 12 shots
84. Vitamin c/vitamin c infusions
85. Vitamin d/vitamin d3
86. Vitamin e
87. Zinc/zinc ionophores (chloroquine, hydroxychloroquine, quercetin)
**B.** **Drugs Discussed in the Above Mentioned Subreddits**
1. Anti-Viral drugs (Umifenovir)
2. Disulfiram
3. Semax
4. Racetams
**C.** **Other Remedies/Suggestions in the Above Mentioned Subreddits**
1. Avoid refined sugar
2. Homemade Masks
3. Intentional Immunization Through Voluntary Light Exposure
4. No vapes. Not with contaminated weed or vitamin e, or with flavours that can have contaminants
5. Nothing will work. Do not smoke or drink, get exercise and eat a well-balanced diet.
6. Reuse masks by baking in the oven at 70 °C for 30 min
7. Sleep

The range of supplements/remedies mentioned by Redditors is being provided here; since a quantitative analysis of the supplements/remedies was not a primary goal of the present study, the compounds were here listed alphabetically.

**Table 3:** Main themes summary.

**Main Theme 1. The Lockdown-Associated: Immunity, Psychotropic(s) of Choice and Drug Dosage/Intake Issues.**
Sub-theme 1. Drug use and impact on immunity levels.
Sub-theme 2. Looking for the optimal recreational/prescribing drugs, alone or in combination, to cope with the pandemic.
Sub-theme 3. Drug dosage adjustment and intake modalities’ issues.
**Main Theme 2. Life at the time of COVID-19, drug dealing, drug-related behaviour and post-quarantine plans’ issues.**
Sub-theme 4. Drug supplying and “customer care” issues.
Sub-theme 5. Drug-related behavioural issues.
Sub-theme 6. Post-quarantine plans.
**Main Theme 3. Lockdown-related psychopathological issues.**
**Main Theme 4. Peer-to-peer advice at the time of COVID-19.**

**Table 4:** Categories/Types of users.

**Type 1. Redditors Considering Continuing the Use of Drugs Despite the Pandemic.**
e.g., Sorry, imma gonna keep doing drugs as usual.
e.g., If I’m on lockdown for 2 weeks I’m most certainly not staying sober.; Do acid first, then go sober! I don’t give af about the coronavirus; WH can fuck off (WH = White House).
**Type 2. Couch Epidemiologists**
e.g., (…) I realized the other day that the early warning symptoms of coronavirus are basically identical to mild heroin withdrawals so … now I’ve just been kinda uncertain as to whether I actually have coronavirus or if (…) I just feel better when I hit the black cause it’s getting me well lmao yikes.
e.g., MDMA weakens your immun system. And I’m not sure how it is with amphetamine. The chance of dying by using mdma and getting Corona virus in 1–2 days after is bigger than 0.2%.
**Type 3. Conspirationists/Pseudo-Science Influencers**
e.g., The seed code is CORONA. Enjoy, psychonauts; Don’t worry, the CIA will continue to funnel in all yalls drugs…
e.g., Can you ask the DMT entities to help us solve this COVID-19 situation?
e.g., Psychedelics are the cure.
e.g., A theory for everything.
e.g., memento mori corona … drawn under the influence of psychic tv and coil.
**Type 4. Recovery-Focused Users**
e.g., Despite lockdown, and other posts on here, you DONT have to relapse. This time can be used for recovery instead……
e.g., Guys! Being sober can be really nice, too…
e.g., Ok dad, I’ll stop the molly.

## Data Availability

The sources from which the data presented in this study are derived are properly mentioned within the article and supplementary material.

## Supplementary Materials

The following are available online at https://www.mdpi.com/article/10.3390/brainsci11070907/s1, Table S1: Selected subreddits’ quantitative analysis: members, posts/comments by keywords, grouped by pharmacological classes (vertical) and according to redditor’s classification (horizontal), Table S2: Main Themes, Table S3: Selected subreddits for quantitative analysis, Table S4: r/Drugs related subreddits (as grouped by redditors).
