# Supplementary material for: COVID-19 Pandemic Impact on Substance Misuse: A Social Media Listening, Mixed Method Analysis
Source: Brain Sci. 2021 Jul 9;11(7):907. doi: 10.3390/brainsci11070907 (PMC8303488; doi:10.3390/brainsci11070907)
Supplement: Supplementary file 1 [file brainsci-11-00907-s001.zip › Table S2 Main themes Covid-19 impact on substance misuse psychonauts.pdf]

Table S2. Main Themes.

Main theme 1. The lockdown-associated: immunity; psychotropic(s) of choice; and drug dosage/intake issues.

Sub-theme 1. Drug use and impact on immunity levels.

- **Immune system concerns**
  - *Cannabis weakens your immune system, I believe.*
  - *If youre on high doses of opioids they suggest you get pneumonia by going in to acute withdrawal and aspirating fluid when you throw up. That will prevent COVID-19, since you'll die of normal pneumonia before coronavirus. Good plan, drug warriors....;*
  - *It's recommended to avoid drinking alcohol as it also has a detrimental effect on the immune system.*
  - *i read kratom actually boosts your immune system. I could swear thats why I never get sick lol*
  - *Maybe don't do MDMA or other drugs that mess with your immune system.*
  - *Substances that boost immune response? (…) Im curious in both recreational and non recreational ones. (…) Edit: please provide evidence for claims while making them.*
  - *Anyone know if suboxone effects the immune system much? If the answer is yes don't tell me, I get severe health anxiety.*
  - *I know for a fact that alcohol seriously fucks with your immune system. I also have a suspicion that stimulants are also not good for it just because of the dehydration, lack of eating, lack of sleep, etc. Also, the media is really blowing this out of proportion.*
  - *What if you have AD/HD and use amphetamines daily as treatment? Will this impact my immune system if I stick to the dosages I've been instructed to use by my physician?; Tripping with Schizophrenia (opinions?).*
  - *I realized the other day that the early warning symptoms of coronavirus are basically identical to mild heroin withdrawals so ... now I've just been kinda uncertain as to whether I actually have coronavirus or if I just feel better when I hit the black cause it's getting me well lmao yikes.*

- **Sanitizing issues**
  - *Disinfecting ecstasy (…) Hey, sorry is this is a dumb question, but: is there anything I can do to molly to make sure that COVID isn't on it? Should I attempt to wash the pill, or?*

Sub-theme 2. Looking for the optimal recreational/prescribing drugs, alone or in combination, to cope with the pandemic.

- **Discussed drugs**
  - *What's in your doomsday stash? What would you still like to add before lockdown happens (assuming we end up like Italy)? What kind of activities do you like to do while high to pass the time? Here's a rundown of what I've got: Codeine 30 mg; lots of it. Weed in various forms from the dispensary (3 vape carts, 4 bags of gummies, 2 grams of flower, 2 strains I grew myself, etc). CBD flower & isolate. Kratom; lots of it. Benzedrex inhalers x 2. Caffeine tablets, 200mg. Xanax, 0.5 mg. Norco (as of yesterday, thanks dentist!), 7.5mg x 20 tabs. Fioricet x about 20 tablets. Coca powder & tea bags x 100g powder, 5 teabags. Various random drugs I don't take (methocarbomol, alcohol, guanfacine, klonopin, phentermine).*
  - *Basically snorting 4-FA & eating methylone for days on end & sleeping; I have only a little of shrooms and 7 LSD tabs for me;*
  - *All I'd need is some alcohol. Not gonna list any quantities.....*
  - *I'll just say "more than enough" to get me (and a few friends) through at least a couple weeks of lockdown (…) Acid, ketamine, mdma, 4-aco-dmt, 4-ho-met, 2cb, weed, hash, distillate and empty carts, CBD flower and tincture,;*
  - *LSD's an all-day trip*
  - *DMT, changa;*

- *Quarter ounce of kush Eighth of sativa 50 doses of 100mg psilocybin 300 x 30/500 codeine paracetamol 5 x 30mg fake adderall 60 x 200mg modafinil 1KG of Phenibut 1lb of kratom 8oz kava kava 50lbs of malt for home brewing (…) Dang, I've never really put it on a list before. It can't be normal that I have all this. I don't even use most of it;*
- *38 30mg vyvanse 2 clear thc distillate carts 15 suboxone 8mg; got 48 packs of mango juul pods; 2 people 2.5kg cannabis 7g shatter 1g distillate 1kg trim (spliffs) 60mg adderall, 30 day supply 5mg desoxyn, 30 day supply 200mcg LSD-25, 10 hits 3.5g mescaline 1g dmt 30g amanita muscaria, dried 30g cubensis 2kg espresso 1.5L Hornitos Tequila Blanco 1 2oz XX Cerveza, 30 bottle case;*
- *10oz of Indica Weed, 20 Tabs of Acid, 20g of Shrooms, 4g of MD, 4g of Ket, few 2CBs, plenty of Xanax, Nitrous*
- *For party drugs and whole rave scene the story is different. Ban of public gatherings killed public drug parties. All my fellow ravers share the same opinion - chill the fuck down for now, and celebrate when it's over. Tl;Dr: party drug market is crashing, but mostly due to social distancing. Weed market is fully functional with minor inconveniences.*
- *I work from home right now, so cocaine is next to my workstation. I hopefully have some MDMA coming in because me and my girl want to celebrate her birthday and it's in a few days when everything is locked down. (…)*
- *Fear nothing! Not even a pandemic! I’m still posted up smoking all day.*
- *I personally have bought a lot more weed that usual because all I can do is sit at home and smoke.*
- *Forced to detox off amphetamine because it doesnt get made in my country and every country in eu is on lockdown. (…) Maybe i'll turn into alcohol or weed for the time being who knows.*
- *My advice is turn to weed rather than alcohol. Weed can keep you calm as it may not seem like it at first. And if its good weed it may help you focus. Not the funny weed.*
- *It hasn't really affected me. I am a daily IV meth user. It was really hard to find alcohol wipes for before doing a shot but otherwise no changes.*

- **Mixes/combinations**
  - *Drug combinations and COVID-19 (…) I see reports that all NSAID's should be avoided if you get this COVID-19 but they all seem to reference Ibuprofen.*
  - *Gabapentin + Alcohol (…).*
  - *a bit of weed + a sniff of midma + a bit of acid all while coming up on some that juicy rona fever 😍🤒 stay safe but if you hit that rona make sure to stay hydrated 💕edit: ok i'm not funny this is just a joke about covid-19 fever :(*
  - *Shrooms + white claw + pineapple = a great night (…)*
  - *codeine, wine and weed, and tbh its not bad.*
  - *Any recreational value mixing Modafinil and alcohol?? (…)*
  - *You’d be better off mixing caffeine with alcohol or sniffing glue*
  - *Fun combo Monday! On the menu today? 45mg oxy, 2.5gs of phenibut, Guayusa for caffeine, and some bud :) (…)*
  - *Maximizing beer+weed effects or Getting through Quarentine (…)*
  - *I’m prescribed bupropion (Wellbutrin) 300mg and ziprasidone(Geodon) 20mg both once daily. will I have any adverse effects if I eat a point or two? Quarantine got me bored as hell. Should I also invest in a test kit prior?*
  - *nutmeg with LSD and weed*
  - *I recently did a solo Candy Flip on Sunday during quarantine with around 500ug of acid and 200mg of MDMA.*
  - *200µg (2cb EDIT)+ some ket is a good time. I would wait until you're settled in your LSD peak before doing K, and some more during the comedown.; depends on what type of K*
  - *Phenibut + caffeine + weed + xanax is fucking awesome (…)*
  - *The following is what I've taken along with the 40mg oxy (after searching the entire housefor anything i can use); 1 x Fentanyl patch 50micro/hour (applied to my chest) 3 x 50mg Lyrica capsules 2 x 25mg Phenegarn (Promethazine Hydrochloride) 3 x co-codamol 30mg codine / 500mg paracetamol*
  - *Anybody else ever make “potions”? (…) Examples would be ground klonopin in Kratom tea, maybe a splash of vodka or something. Wine, vodka, beer, vyvanse all mixed together. Etc, etc. Quarantine has clearly driven me crazy.*
  - *tequila vodka powdered Adderall and focalin with a tab soaked in it*
  - *My weed dealer when I was younger used to make a little concoction he called the Bargarita. He would make a regular margarita. About a 12 ounce one, but with a twist! He would take like 2-3 Xanax Bars and grind them down to very fine powder then dump them off into it.*

- *Sometimes we would get our hands on a syrup called Rezira. Take that syrup, mix it with Jolly Rancher drinks, and 2 or 3 shots of vodka. We had pure powder caffeine back before it got banned. Sprinkle a pinch in. Caffeine, ephedrine, smidge of the hair of the dog, and hydrocodone*
- *A long time ago I put crushed up Adderall XR - the little balls in the capsule, taken out and crushed to powder - into a shot with whiskey. I stirred it and watched it dissolve. realized it would taste awful, so I added some coke. As the bubbles settled down, I watched, NO BULLSHIT, as the little XR balls reformed.*
- *Pour some red bull into the pyrex a couple drops of some etizolam solution 100 mg tramadol crushed up and stir it up and pour it into a cup made me feel like a wizard!*
- *I did mix caffeine, speed energy drink and put it in my shaker so I could drink it in school*
- *Third experiment with gabapentin. This time mixed with 1P-LSD. (…) I assume- have been a bit annoyed due to the quarantine and feeling this good made me experience it as something even better than it was.*
- *Benzo+ food addiction+ quarantine= I want to fucking kill myself (…) dose 3-5mg of etizolam*
- *Lemon tek 2g of some good mush, smoke a phat bowl on the peak if wanted, then wait for almost the entire comedown then roll .15 of some mdma.*

- **Drug starting/drug suggestions during Covid-19 pandemic**
  - *I was considering getting into micro dosing yeah. Also maybe trying lions mane and NAC?*
  - *I am bored in quarantine and I am out of weed and benzo as my dealer got coronavirus and stopped delivering. I am looking for another way to get high. I have lots of Lexapro and was wondering how many mg I should take to get high. Never had lexapro before.*
  - *So, coke and essay writing? Does that work? Anyone have experience? (…) Thoughts? I've also go K, md, and some weed, but figured coke would be my best bet. I'm open to suggestions because I'm bored as hell.*
  - *I'm home from college early due to the Corona Virus and my parents don't won't me leaving the house so understandably I'm extremely bored. Now normally at school I would just smoke and be chilling but I don't really want to smoke in my room because of the smell and my parents don't really like weed. So I was wondering what otc alternatives might produce similar effects or really anything to keep me entertained.*
  - *So I've been mentally preparing to do LSD for a few months and I've been ready. But with the whole corona, that's not gunna happen soon for me. Im at home now and my sister is open to me doing shrooms, however.*
  - *Cant smoke in my house when parents are in, need a non smelly drug*
  - *How to keep drug use under control? (…) Hey people.. all this free time during quarantine has me thinking about tripping quiet a lot, definitely more than usual. That's why I was wondering what kind of guidelines you have in place to make sure you don't over do it. Like certain time spans between use, dose limits, only for special occasions etc. Let me know for which substance in particular or just in general*
  - *I've been trying out the naropin and it's pretty nice. I've got 3 bottles still so it should last me through the quarantine. I'm not sure if I wanna try the fent patches really. I may give it a shot for the one time.*
  - *How to desalinate cooking wine? (…) Well... quarantine has me completely out of everything, and the only thing I have is some white cooking wine. I know it's drinkable, but is there a way to get rid of the salt? Thanks in advance!*
  - *DMT, Shrooms, Ketamine, Edibles/Weed, Opioids, Benzos, Cocaine are all good indoor drugs.*

Sub-theme 3. Drug dosage adjustment and intake modalities' issues.

- **Asking for dose adjustment tips during quarantine/lockdown**
  - *taking double dose ritalin (NEED ADVICE) (…) dr said i can take 2 of the short acting ones. one in morning before work and one after work for homework my 5mg doesnt work anymore and my states on COVID-19 lock down so i cant go to the dr for a better option. can i just take two of my short acting meds? i just wanna get my final done!!!*
  - *Will i feel anything on 95 mg od codeine? (…) so i have A couple of codeine phosphas + sulfgogaiacolum shit will this get me high? I'm just so desperate cuz covid makes everything so hard to get*
  - *I can no longer workout without getting high (…) However due to the Covid-19 lockdown, I've had to workout at home. Being at home has led to me getting high on the reg, and before working out cause it feels AMAZING. (…) I don't feel the need to take the Tramadol generally, but to workout I feel like I need it.*

- *So I just got out of being in the hospital for 2 days from pancreatitis I got from drinking too much during quarantine.(…) besides weed, which I already smoke, what is a good alternative out there that I can take when out with friends that would make me relaxed, sociable etc.... I was thinking about kava since it's popular where I live and can have that social aspect as well when you're drinking it in a group*

- **Quitting/withdrawal/tapering/prescription issues**
    - *Nicotine withdrawals weren't too bad*
    - *How did you feel coming off antidepressants? Any side effects? Anxiety? Nausea? (…) The stuff with Covid-19 isn't help either. I just feel weird and I'm wondering if it's because I've lowered my dose...*
    - *Who's relapsed or seen their drug use skyrocket since being put on lockdown/quarantine?*
    - *Thoughts on Kava Kava? (…) So I quit my heavy usage of cannabis and moderate usage of LSD about two months ago when Australia began a COVID lockdown (also my dealers was across closed state lines, making it impossible to pick up from him) and used the opportunity to sober up (…)*
    - *How long does Morphine IM last? (…) My questions to you is how long does 100mg morphine IM work? I am using it to get off heroin and stay stable on methadone and start lowering. I cant lower methadone because of coronavirus scare. I tried 3 times this and past week.*
    - *ADHD medication (…) Does anyone know if doctors are allowed to mail the prescription in the mail so I can take it to a pharmacy here? TIA*
    - *I need help quitting Oxycodone (…) I smoke a lot of weed and that HELLA helps distract me. Plus in the wake of coronavirus I have no job for now so I have time to kind of go into a sort of self-rehabilitation.*
    - *Trams anyone? (…) I have no access to my other vices except trams. (Yes quarantine is taking its toll.) I'm not to happy about it. (…)*
    - *Quit Heroin due to quarantine (…) Don't applaud, I'm still using all kinds of other shit.*
    - *Quitting Smoking (tobacco) During an Impossibly Stressful Time (…) I've been trying to keep myself distracted, but I honestly wasn't prepared for the intensity to which my depression has worsened as a result of this.*
    - *Venlafaxine (effexor) question for anybody else taking it (…) My pharmacy has been closed because of lockdown until today, so I've missed about 5 days of doses. But I haven't once felt this happy or energetic ever since taking it, i've got no symptoms of withdrawing, I feel alive again, and my anxiety and stress levels are lower. (…)*

- **Changing ways of assumption**
    - *Too anxious to smoke up... (…) Concentrates and herb vaping should be easier on your lungs just empty the joints if you have a vape. Also oral and sublingual methods, edibles/tincture/distillate*
    - *Maybe a good time to switch to Edibles?*
    - *I can't smoke at home during quarantine so I was wondering if there are any other methods to consume the nicotine in my cigarettes without smoking them. (...) Maybe you can chew the tobacco (…) grind it super fine and snort it like nasal snuff. (…) Soak one in water and (slowly) drink the juice (..) You could break up the ciggie and stick the tobacco in your lip*

Main Theme 2. Life at the time of Covid-19; drug dealing; drug-related behaviour; and after-quarantine plans' issues.

Sub-theme 4. Drug supplying and 'customer care' issues.

- *My dealer just dropped off a care package (…) But he came back with a whole care package for me, lsd, shrooms, shatter and dmt... The man knows what I like... Tells me he knows that times are tough, that being stuck at home sucks and he hopes this helps and didnt ask for a penny.;*
- *the drug gang I'm sourcing my material from have organized to help combat covid more than local authorities did (…) I had to visit today to stock up on some drugs because of the ongoing lockdown only to be surprised that they mobilized their gang to operate according to the safety regulations needed to prevent COVID-19 contamination, every gang member had masks and gloves and the drugs are double prepackaged and the people are obliged to respect the line with 3 meters between each customer;*

- *(…) Its different I guess depends on personalities & situations, some had this COVID issue made them abuse drugs while others managed to quit.; That being said, I haven't noticed any changes in the drug market. A few people I know have mentioned that their plugs raised prices a bit, but it wasn't enough that they had to raise their prices (as long as they're buying in bulk they can still make money). People are still willing to meet face to face, make deliveries, and everything as if it were any other day.; That's strange that the price goes up. In here some product price was cut in half because the demand is real low. I'm glad I'm not into that thing or else I would be fucked lol;*
- *Mail order isn't risky in Canada; I live in the Southeastern United States. Also, any mail-order requires me a) getting a bank account during all this b) depositing the little cash I have c) ordering a package to my mother's address and d) securing said package before she does. The process is fraught with risk.*
- *They've gotten more expensive where I live*
- *Party drugs gonna get scarce? (…) I'm thinking the black market for party drugs is gonna significantly dry up here for the next several months in the US as this coronavirus lockdown is gonna likely be extended into the summer... Thoughts?*
- *Not just party drugs, pretty much every drug except weed needs to be imported either as a whole or as precursors*
- *Kiwi here. My main source who is usually super reliable just can't get coke due to mules not being able to get in and out of the country. Quelle horreur.*
- *Enough weed for months - SO happy 🌼 (…) Background: I suffer from schizoaffective disorder and am prescribed heaps of medicine to little effect, including Xanax, abilify, and lyrica. I'm going through a major life crisis which is heavily exacerbated by the whole coronavirus thing. I've over the years found, that the only thing that calms my symptoms, everything from depression, suicidal ideation, anxiety and mania to psychoses, is weed. (…) The whole drug market here is turned upside down since the partial lockdown set in, and I haven't been able to get good weed to calm my panic disorder and suicidal thoughts for a while, which has stressed me the fuck out. Additionally, dealers over here are ripping people off with fake shit left and right. I have mild symptoms of corona, so I've been in quarantine for six days. I imagine that this corona lockdown will stay out for months to come. For a week, I've looked everywhere and put all my hustling skills to max use to try to find some weed. Last night, it happened: I locked down a deal to get me enough to sustain myself for anywhere between three to six months.*
- *Where I live, supply and demand have both gone up. Shit's popping off more than ever but they have no problems keeping it stocked.*
- *well basically all of the suppliers i know struggling to find someone to g p's of bud is a large enough sample size for me to decide that it is more difficult to get bulk due to corona*
- *I agree the virus has already and will continue to make it harder to get weed, but not because of state borders being closed. Its also because everyone is staying the fuck inside and distribution networks arent working as they normally would. Also demand is be much higher because everyone is home watching Netflix all day.*
- *i always see majority of the dealers i know having the same strain of weed, i assume this is because it all comes from the same location. also i don't think that there's a shortage, simply suppliers will need to find a new source for their bulk*
- *They closed coffeeshops (that's what we call places where you can buy weed in the Netherlands) for about a day here, then when they saw the huge lines and realised everyone will go to the black market, they opened them again...*
- *Postal service and corona (…) Yo guys im from southern europe(Balkans) got shipment waiting for in NL and its almost 2weeks by now, do you think it could be corona virus delayed shipping process and stuff. Or is it normal to wait this long. I got party coming up and kinda relaying on shipment so...*
- *is it weird to make baked goods for your dealer ? (…) im buying something from a dealer sometime this week . the quarantine made it hard to find someone to sell me stuff, but i found someone through a mutual friend . i've never really did a drug deal type of thing and i don't really know what to do . i feel bad for having him get it and drive it all the way to me*
- *My quarantine weed stock is at the end and I won't be able to re-up . I started having dreams about weed and I'm kinda scared of losing my mind. Any advices?*
- *I think the biggest impact will be on supply chain. Will dealers stop selling? Definitely not, that'd mean giving up a source of income in a time of high unemployment. The supply chain thus spreads the virus. Lockdowns and quarantines will not deter addicts.*
- *Royal mail are still working so our drug supply will not be affected much if at all.*

Sub-theme 5. Drug-related behavioural issues.

- **Generic quarantine/lockdown times behaviours**
  - *I'm gonna use this quarantine to get sober and hopefully get back to just socially drinking/using*
  - *I'm grateful for my boyfriend, Xbox and a daily altered state since we're quarantined.;*
  - *Bored and stuck inside the house due to COVID-19 so I took some leftover hydrocodone from a surgery I had a few months back since I can't get weed lol.*
  - *(…) he revealed to me he's been regularly using meth for 6 years. because of covid, his use has gone from "functional" to going on more binges. he's even started dealing (…)*

- *The virus has really been picking up in my state, and I wanted to see how my fellow users are doing. Personally, I haven't changed much, but I'm thinking about stocking up on a few things like benzos and staying away from substances that weaken my immune system.*
- *Seeking advice for my next shroom trip (…) The problem that I run into is the setting. I live with my family (21) and (as irrational or not as it may seem) I need whatever setting I'm in to be free of anyone else's energy, even if they're in rooms separate from mine. (…)*
- *Stealing pills (…) I normally don't steal anything, save for a couple times I took some hydros and xans from an abusive uncle. This time I discovered two full bottles of oxy and xanax. (…) Maybe 15 oxy and 10 xanax. Nothing noticable, there were maybe 100 of each. I fucking hate myself for it. I am not proud of this behavior I've developed. This habit. I am not an addict. It's a really, really shitty habit and I have no clue why I do this. With corona going around, I have no job and am basically broke. My stash was running low, I panicked, I saw an opportunity.*
- *How quarantining, social distancing, living off stimulus and unemployment has affected me (…) I moved in with my sister who is down to help me and I've had to withdraw. Well I bought ten xanax, multivitamins, magnesium, potassium, l-tyrisone an eighth and just take an quarter piece of xan every few hours smoke and I feel great. Sip a cup of coffee and smoke some bud and think. I was stuck in a loop remember Einstein's theory of insanity. I mean we're all stuck in some kind of loop but make it a positive one.*
- *Took a brief intermission from drugs that I was hoping would last longer but this whole quarantine has got me sucked back into habitual use.(…) Im prescribed gabapentin and lunesta right now and have gotten very close to losing any cravings for it up until quarantine. (…) It probably doesn't help that in my boredom I've started collecting novel drugs.*
- *So, because of the quarantine there was a month delay for the weed I ordered to arrive, which meant I was going on a forced TB. I was of course bored to hell, having to be sober 24/7, so I decided I was going to get 'naturally' high through meditation, something I practiced in the past but have given up in the recent years. (…) But now when the weed I've given up on unexpectedly arrived and I stared smoking again it's always consistently great, purely because I've taken control of my mind through meditation, I can avoid going down those negative thought patterns- which used to put me in a bad and paranoid mood.*
- *I think with quarantine, many things I enjoy more whilst sober I can't do. One thing I can do though that's more fun when sober is playing competitive games*
- *During quarantine I am finding out that smoking a little before a workout is an amazing experience. I go to a calisthenics park, and it's even better if the sun is out and you put on some good album. It becomes a whole different experience, along with a soundtrack. Now I am writing this, coming off of the high, and the post-workout endorphins create a wonderful synergy with the cannabinoids.*
- *Bong in Shower (…) Edit: I'm asking bc parents are home and only viable place is bathroom*
- *Most people enjoying quarantine, doing drugs, sleeping, playing games. I'm in quarantine with a baby, haven't done shit for myself in 1.5 years 😅 Enjoy your times people, once you become a parent shit starts to get real*
- *Please tell me I'm not the only one this desperate (…) I scraped my grinder with a pocket knife for an hour and rolled a spliff that tasted like plastic. Due to my low tolerance it kinda worked though. These quarantine times got me doing desperate shit*
- *(…) moving heavy shit in general constantly and waking up early. I am so exhausted and my parents do. Not. Care. I do not know how much more I can handle! The only time i rest is at night, when it pours hard, when I have homework on weekdays. Fucking Tramadol is keeping me from being another victim of the pandemic, god bless😭*
- *I was going to get sober but then the coronavirus happened.*

- **Various thoughts during quarantine**
  - *Realization while high (…) Do me a favor, if you smoke/vape any drug go to a enclosed room and close windows and shut off fans. Take a hit and exhale like you would when you breath normally. See that vapor? Spreading out everywhere. Imagine that vapor is COVID-19. (…) Stop treating this shit like a fucking god-damn meme. Save the memes for after the outbreak but right now we are fighting a war. The difference is that people don't see the casualties and think it doesn't apply to them.*
  - *Everyone is doing drugs during this quarantine (…) And I'm stuck at work everyday 9+ hour shifts because I work in healthcare. (…) Almost every post I see on reddit is a picture of drugs and the caption "enjoying some _____ during quarantine!" Get fucked up for me y'all.*
  - *The Psychedelic Enlightenment (…) Any form of meaningful exchange is such a big plus in my life in the shadows of COVID. (…) I am not into conspiracies, but I can't help thinking about e.g. DMT being illegal around the world.*
  - *I'm depressed and drugs don't seen like an answer anymore, I don't see the point in doing anything (…) With the covid thing I can't go out home to do shit so I'm stuck in my bad thinking if any of you have went through something close to this and how it went after. (…)*
  - *i can't concentrate without coke (…) plus at the minute everything is slowed because of covid, even the private therapists i was planning to see aren't providing anything other than phone call services, which i'm not about to pay £30 for each phone call so someone i've never met can try and analyse me over the phone. so i'm stuck. (…)*

- *Currently on 70 mg 3-FPM insufflated and 60 mg of O-DSMT ingested after half a hour. Besides the burn, 3-FPM is great, cleaner than amphetamines which I had in at least two occasions 85% pure. My OCD seems to draw back. I feel bathing in dopamine and besides living a shitty period (job lost due to covid, lots of broken dreams and worries about drugs still far from being socially accepted and decriminalized) I feel happy. (…) None can explain how important is for someone living with a mental illness in a poorer western country to have a few hours of well being. (…) Edit. I did two moneky shoulders (whiskey circa 55 mL) and after 3.30 hours redosed 45 mg 3-FPM and 34mg 2 Methyl Ap 237 (which I like more than O-DSMT). Walked a lot took pics of the sea and enjoyed this day a lot.*
- *Comedown dk what else to say (…) I'm moving out, evicted bc of covid. I was sober for MONTHS but I relapsed and I'm ashamed. Idk what to do I'm so depressed I feel like the sky is falling. I'm not homeless. I'm lucky. (…) I'm 22 and my goal is to break the cycle, but here I am addicted, acting the fool. I'd love advice*
- *I heard some random dudes in town where cutting it with blood samples in some drugs and or coughing at them so customers could get infected like in meth and heroin could that even be possible?*
- *Hi! Yesterday it was was mariclous day on shrooms I made a Vaccine to treat the corona and is working! is wonderful how creative you get on shrooms.*
- *(…) Rant: Im just worried about the coronavirus in jail tbh.. It pisses me off theyre doing so little with such a serious virus going around. (…)*
- *Think about your National Health Service please (…) You might think you know what you can handle but right now is not the time to risk it. This is for your benefit too*
- *Being bored as an addict is dangerous (…) Thanks to corona virus I can't go to school or work. This leaves me with literally nothing to do except for a hobby or two, which doesn't mean much. Now there's barely anything to keep my mind off my depression and how much I hate living in general. I think I'll just smoke weed til I pass out for the next 2 weeks. Hopefully I don go back to hard drugs cause now living without them is EVEN FUCKING WORSE*
- *(…) If I can't escape my fam or receive a fuck ton of psychs I'm gonna be mentally broken. I have the money and know dealers, but corona making people stop delivery. Corona might kill me with Xanax. Fuck this. (…)*
- *Nicotine is the best (…) Having trouble getting drugs in quarantine, so I don't smoke cigs for 12hrs then smoke one to get high from the buzz, and it's fucking great*
- *Memantine: The perfect quarantine chem! (…) Took 70mg a couple hours ago, expecting to be disso'd till friday! Won't be sleeping anytime soon, but music sounds wonderful!*
- *opiates are the ultimate escape drug for me. quarantine sucks lmao*
- *MDMA really is amazing, I can see exactly how people get addicted to it. (…) One good thing about quarantine is all the club's shutting and it's forcing most people to give it a much needed break till it's over, but I know exactly how it's going to go when the club's reopen.*
- *Love letter to Gabapentin (…) Gabapentin is so tragically underrated. Staggering doses is tedious, but it's 1000% worth it in the end. Despite what you may have been told, fatty food and carbonated drinks aren't necessary for full effects. I'm sitting here, 2 grams staggered over 4 hours, absolutely glowing, warm waves of contentment rolling over me. I keep smiling like an idiot. I can talk to anyone about anything and feel no anxiety. I've fallen in love with this drug. <3 Quarantine isn't so bad after all*
- *yeah I've also surprised that as quarantine has progressed, drugs actually started to accumulate to my stash.*
- *Nice haha, yeah I think this quarantine has affected everyone for better or for worse.*
- *Being sober is fucking boring. (…) During this damn quarantine i remember just a few months ago i had an abundance of weed, now ive been forcefully sober for at least 3 weeks. (…) Fuck my psychiatrist for not giving me any Xanax.*
- *Who up and just needa vent about quarantine bs? (…) There's just been so much goin on during quarantine and I'm just a lot of people are turning to substances to take up their free time so I just wanna hear about other people's struggles*
- *Since I've been in quarantine I've been smoking more by myself and before I it I genuinely liked it more by myself. But When ive been smoking lately I've been getting super depressed and self conscious coming from a mind who already has low self esteem.*
- *Research chemical clonazolam / overdose (…) Been taking for a couple months, I hsve benzo addiction so it's nearly impossible to monitor my use. Is go through 6 mL over a few days. But quarantine and heart ache, I drank a whole bottle over the coarse of a day. Luckily my friend came over that evening and was worried for how slowly I was breathing. Trip to the ICU and psych ward , now I'm trying to remain sober from that drug.*
- *I really really like adderall (…) One of my dealers was selling addy tho and since it's quarantine and I'm bored I figured why not, I got some work I need to do anyways. So I popped one tonight thinking I was gonna bust through a project but holy shit I was just taken over by euphoria.*

- **Tripping during quarantine/lockdown**
  - *Friend tries to commit suicide and stolen gucci glasses in LSD *HORROR* TRIP (…) The police arrive and they are coming in wearing gloves and mask, cause COVID-19, I get shit scared, and think this is the end of the world.*

- *I finally wrote a report on DMT (…) I was stricken with worry about their fate amidst the COVID-19 pandemic. I began to project how I would feel if they were to pass from it, to empathize with that potential future trauma.*
- *LSD has completely discarded the pain of my depression (…) During the covid epidemic I spontaneously decided to take acid and with little to no expectation, ive come out of this with the most effective results I've ever had over a highly neutral trip. (…) Nothing special happened but I've noticed the thoughts that would make me depressed and place me into a depression no longer emotionally affect me. Or affect me at all for that matter. (…) The specific thoughts still come around here and there but they bo longer effect me whatsoever and as a result begin to occur way less often. I'm extremely thankful for this and am glad to be able to experience this (…)*
- *Trip report - xx mg 2CB (…) T 0:45 - Something kept buzzing next to me, drawing me away from sensory bliss, so I took off my goggles and saw that my country was infected with the coronavirus and cases were spreading across the world. I wasn't sure if this was reality, but either way, I didn't want to be here anymore, so I took another puff of weed and left the world. Time and space melded into a backdrop of VR visuals. (…)*
- *Worst time to trip (…) Got crazy drunk last night, took a heroic dose of acid, throwing up on acid is rough, throwing up thinking you have corona virus is the absolute worse thing ever. Still tripping, life is definitely weird rn*
- *Trip Report: A Spontaneous Quarantine Ketamine Rave (…) This is a story of my favorite ketamine experience to date, that happened one week ago today. I had multiple bumps and lines for a crazy spontaneous rave party that lasted until 5 in the morning. (…)*
- *This time I learn what MDMA really is. (…) Fast forward to today the plug hit me up and told me he was trying to get rid of the last of his molly and dropped the price , I had some bones and decided to treat my self during this oh-so lonely quarantine night. (…) Molly is definitely at the top of my list now. (…) TL;DR:First time rolling was ruined by the nose candy. Second time minus the blow, orgasmic.*
- *Terrifying trip during COVID-19 lockdown in London (Trip report) (…) during the COVID-19 quarantine I have been finding myself doing mini-trips at home / outside in parks whilst practicing social distancing and I must say it's been very nice. A friend of mine hit me up after about my 2nd trip during this quarantine and wanted to join in on the fun, lets call him "Andrew".*

Sub-theme 6. Post-quarantine plans.

- *Im 16 years old and have smoked weed taken edibles (like 50mg) I've also done shrooms twice going to do acid and Molly after quarantine do u think I could try coke or is that bad for someone my age to try it*
- *How to Structure Multiple-Drug Trip (Weed, LSD, Shrooms, DMT, E) (…) Me and a couple of friends are looking to go on an Airbnb trip post-quarantine (thinking of 5 days, not sure though) and we're wondering how exactly we should structure our time.*
- *What's your plans for once lockdowns loosened or over in your country? (…) What drugs do you plan on taking that you've not had for a while, new drugs to try or what's your favourite combinations your looking forward to having again*
- *Molly and Lexapro (Escitalopram) (…) I know there's the chance of serotonin syndrome but has anyone actually taken molly while being on any type of Ssri? I've also heard it could dull the effects but I'd love to hear some experiences cause I really wanna roll once this whole quarantine shit is done*

Main theme 3. Lockdown-related psychopathological issues.

- **Generic psychiatric issues**
  - *Did anyone recently get a sore throat after snorting coke? (…) I'm wondering if I had Covid or some other illness.*
  - *a week after I got off the Methadone, the whole Covid-19 crap and other stuff in my life whacked me with hella stress, anxiety, and palpitations. Soooo I'm back on the dope. :/ just trying to not lose it completely. What I need is to find a psychiatrist and counselor*
  - *Anyone gets ticks or weird compulsive behaviour after getting clean off percs/fent? (…) I'm just wondering if anyone else has experienced this or if its stemming from my anxiety or quarantine or whatever. TIA*

- *Blocked ears after coke! (…) i have had minor nosebleeds before but never blocked ears.. And it all began after i bought the first gram POST QUARANTINE. I am thinking the coke im getting is too cut because im also feeling too shaky after a few lines? Its a bummer cause this dealer had reaaally nice blow but now I feel like hes cutting because he cant get much as often because my country (Paraguay) is having major strict controls in the streets, specially at night due to coronavirus laws. (…)*
- *During this quarantine the bigger issue is how it's affecting mental health compared to the actual virus itself, I wonder why?*
- *Quarantine Blues (…) Since quarantine began, Ive just been abusing the shit out of drugs. We are on the 10th week now, for 9 of them I've been going hard. GHB, Clonazepam, etizolam, soma and pregabalin are the ones im concerned about. I've also done a lot of MDMA, methaqualone and kanna. I had been off everything prior to 10 weeks ago, I had my doc put me back on 1mg Clonazepam a day. I then began using GHB all day during the day, and taking my clonazepam at night. As lockdown boredom increased, I started getting crazier with my nights. I would take G into the night, then knock myself out with a combo of soma, Clonazepam and Etizolam. Then I got in pregabalin. I had never done this drug before....and wow.*
- *I have all the same things in my mind every day actually. I want to take some drugs but trying prevent me from this. Also i'm in mental struggle with myself that borders with the depression.*
- *Borderline psychosis-feeling on Zoldorm (…) Background, I'm a 17 year old guy from Europe. Now as the covid-lockdown is going on I started to feel really bored. Smoked all my weed a few days prior to this and got nothing else to do, so I had the great idea of taking some Zoldorm just for fun. I took one 10mg pill (no tolerance to Z-stuff). (…) A few minutes later everything pretty much went batshit-crazy. (…)*
- *opinion on taking antidepressants on your own without consulting a psychatrist? im really at my end here nothing really helps and i dont have access to therapy or anything because of the covid situation but also because the mental health services suck here. idk if i should just try taking them.*
- *Smoking Codeine? And onset of action? For a serious reason (…) I have bipolar and have just hit my depressive episode again, i'm on meds but it's not really enough and we can't update because of corona so it's just a matter of something slightly bad happening or even just boredom and i'm fogged and find myself taking boxes of pills and alcohol (did not happen before but i did choke myself or burn which parents intervened, and trimebutine, yes that weak thing, intervened it many times but i doubt you can smoke it) so i can get naproxen codeine without a prescription and extract the codeine, or so i saw, i can right?*

- **HPPD**
  - *I did LSD at the start of the quarantine with a friend. Now sometimes I still see some hallucination with shadows or with some building is it normal ?*
  - *Went for a walk, normally don't exercise. The entire road started breathing. (…) I'm not sure if this is HPPD or extreme stress related to the fact that I work at a hospital during this coronavirus thing. Just wondering what some of you all think/ have experienced.*
  - *HPPD keeps giving me a shit ton of anxiety, advice? (…) Now that i'm in quarantine i can't stop thinking about it.*
  - *HPPD and history of schizophrenia hearing voices (…) Worried I could be slowly breaking into a psychotic episode although I feel fine if a little down from quarantine.*
  - *I have been using CBD oil sometimes which contained 0.2% THC and was full spectrum and I was fine. It made me really relaxed but not high at all. I also vaped isolate CBD. Though CBD increases my visuals it makes me comfy and has a positive effect on my overall well feeling and anxiety. (…) I ordered some CBD weed which is 8% CBD 0.2% THC coming in Friday. I have Clonazolam on hand which I really take with care and moderate doses only (1 time 0.25mg in the last 2 weeks)*
  - *But since all this Corona thing I keep hearing my own voice in my head like narrating my thoughts constantly - now I already suffer from Anxiety and Depression (specifically Health anxiety) and I am very worried that I have something like Schizophrenia instead of HPPD but obviously I'm not sure and it could just be because of how anxious I am at the minute.*

Main theme 4. Peer-to-peer advice at the time of Covid-19.

- **Misleading advices/related comments**
  - *(…) For all these functional addicts stuck in their home, their drug stash is now always just a few steps away, they can snort lines on their desk while their code compiles, they can nod out without fearing that someone will notice. Anyway, if you find yourself in that situation, remember that these are not normal times and it's probably fine to get fucked up at home for the next few weeks*

- *A cocaine only diet provides all the nutrients you need. Food is just a burden for the weak.*
- *Hey, thanks. What do you think about a splash of alcohol on top of MDMA? 😂😂you know some can't help it at times ¯_(ツ)_/¯*

- **Harm reduction**
  - *Stock up and use with caution dummy;*
  - *Before you take a heroic dose or try a new drug/batch...(…) Please make certain what you are doing is safe. Hospitals are more overloaded than usual due to COVID-19. Keep in mind the following: (…)*
  - *Substances I recommend to avoid: Benzodiazepines (Valium, Xanax): Increased risk to pneumonia. Alcohol, Opiates (Heroin or most pain killers), Stimulants (Cocaine, Amph., MDMA): Suppresses immune system. Substances I recommend to pass the time: Be smart. Dont take when sick. These just seem to have the lowest risk factor profiles if you know what your doing. Psychedelics: (Cannabis - edibles or vape, LSD, Ketamine, Shrooms or Mescaline) Peptides: Noopept Tranquilizer: Phenibut Herbs: Kratom*
  - *It's been stated a lot that suddenly stopping drug usage (in light of coronavirus) can have extremely negative effects, which is good and true, but I haven't seen people talking about how if you stop using drugs/greatly decrease your dosage, especially opiates, you should never go back to your old dosage immediately. Your body doesn't have the built up tolerance, and a high dose, even one that you could easily take before your break, can kill you.*

- **Alternative therapies**
  - *Dopamine Fasting Challenge | Meditate for Peace, Health and Happiness | Develop Monk-Like Discipline and Peace of Mind During These Anxious and Uncertain Times. Wishing everyone all the love, joy and blessings (…) Day #1 - Join me in the Dopamine Fasting Challenge during the COVID Self Isolation!*
  - *learn to lucid dream so you can trip for free at night. You can also smoke weed in the dream.*
  - *Try holotropic breathing*
  - *I'd recommend listening to the Joe Rogan episode with Sam and Dan Harris. (…) I also recommend learning the Wim Hof method*
  - *Exercise mainly just stay as busy as possible; I find drawing can help a lot. If you're not that great an artist (like me xD) then adult coloring books help. I know it may sound boring but once you get focused on it time starts to fly.*

- **Reddit Automoderator/BOT posts**
  - It's spelled serotonin, not seratonin. I am a bot, and this action was performed automatically. Please contact the moderators of this subreddit if you have any questions or concerns.
  - It looks like your post mentions suicide. Sometimes, people post questions on r/Drugs when they're not feeling right about their life, and sometimes we're not the best place to ask or provide support. (…)

- **Links and online surveys**

- **Discussed social networks**
  - *If you're on Steam by any chance ask me as much shit about this as you want, I can use DMs if that's a problem*
  - *My source left town and and this quarantine has me so damn bored. I don't know how to ask anyone else, I don't know where to look. Can't ask any of my friends - they are so straight laced and I'm not a clubber. Is there a method to meeting new connections? How do people find connections?*
  - *Grindr or Facebook (look for dudes who are self employed and went to the school of hard knocks lol shitty but true.*
  - *(…) Snapchat*
  - *Really alot of this comes down to Google deciding they know better than the rest of the world and filtering the search results, started a few years back. That's the root of the problem, it's why I shifted most of my research to the darknet lately, or if must be clearnet at least use duckduckgo, no censorship going on either of those places.*

- *Lots of forums with trip reports and info, it's pretty much the same as the clearnet minus the censorship of Google/facebook/YouTube etc. Dread, the darknet version of reddit, always a good place to start.*
- *Point taken. It's how I feel, I'm worried for our society's next 20 years of drug use based on the posts I see here.*
