# Supplementary material for: COVID-19 Pandemic Impact on Substance Misuse: A Social Media Listening, Mixed Method Analysis
Source: Brain Sci. 2021 Jul 9;11(7):907. doi: 10.3390/brainsci11070907 (PMC8303488; doi:10.3390/brainsci11070907)

Table S4. r/Drugs related subreddits (as grouped by redditors).

Table S2a. r/Drugs related subreddits (whole list).

Drugs A-Z (50 subreddits)	members 11/12/2019	members 11/05/2020	% change
**/r/5MeODMT**	1863	*2733*	46,70%
**/r/1P_LSD**	10535	*13184*	25,14%
**/r/2cb**	*17133*	*22212*	29,64%
**/r/4acodmt**	1703	2236	31,30%
**/r/adderall**	34775	*42896*	23,35%
**/r/afinil**	*22947*	*23374*	1,86%
**/r/alcohol**	*69331*	*74183*	7,00%
**/r/ambien**	*65191*	95273	46,14%
**/r/aMT**	419	487	16,23%
**/r/anabolic**	1848	*2028*	9,74%
**/r/Ayahuasca**	*24665*	*29227*	18,50%
**/r/benzodiazepines**	*42140*	*52695*	25,05%
**/r/cannabis**	*95481*	109485	14,67%
**/r/CannabisExtracts**	72511	*83064*	14,55%
**/r/cocaine**	*52934*	*66890*	26,36%
**/r/Cigarettes**	*29037*	*35574*	22,51%
**/r/DrugCombos**	1022	1244	21,72%
**/r/dissociatives**	3838	4742	23,55%
**/r/DissonautUniverse**	1357	1650	21,59%
**/r/DMT**	*128395*	157647	22,78%
**/r/DPH**	4918	*8417*	71,15%
**/r/dxm**	*22300*	*28370*	27,22%
**/r/fentanyl**	*1976*	2464	24,70%
**/r/Kava**	*9892*	*11589*	17,16%
**/r/ketamine**	*25801*	*34282*	32,87%
**/r/Kombucha**	*35487*	*45932*	29,43%
**/r/kratom**	*83084*	92846	11,75%
**/r/lean**	*17463*	*23883*	36,76%
**/r/LSA**	*5544*	*7162*	29,18%
**/r/LSD**	*327175*	422806	29,23%
**/r/MDMA**	*95371*	118103	23,84%
**/r/MemantineHCl**	896	*1155*	28,91%
**/r/mescaline**	6577	*14791*	124,89%
**/r/meth**	*15830*	*21953*	38,68%

**/r/modafinil**	11971	15633	30,59%
**/r/MXE**	*585*	*710*	21,37%
**/r/noids**	2813	*3428*	21,86%
**/r/Nootropics**	*206148*	222373	7,87%
**/r/opiates**	*88356*	*98812*	11,83%
**/r/PCP**	733	1060	44,61%
**/r/phenibut**	*23669*	25374	7,20%
**/r/PoppyTea**	*4638*	*5219*	12,53%
**/r/PsilocybinMushrooms**	21000	*29948*	42,61%
**/r/ResearchChemicals**	*69465*	*83554*	20,28%
**/r/Salvia**	13766	*17762*	29,03%
**/r/steroids**	*77093*	87122	13,01%
**/r/stims**	55355	*66175*	19,55%
**/r/TheSaviorSubstances**	60	93	55,00%
**/r/treedibles**	*84807*	119910	41,39%
**/r/trees**	1450686	*1553229*	7,07%
**/r/gabagoodness* (see Discussion group)**	2151	3684	71,27%
**/r/MAOIs* (see Discussion group)**	526	864	64,26%
**/r/ObscureDrugs* (see Discussion group)**	3979	*6200*	55,82%
Addiction/Recovery	members 11/12/2019	members 11/05/2020	**% change**
Alcohol (10 subreddits)			
/r/alcoholicsanonymous	***	***	***
**/r/alcoholism**	27261	*31447*	15,36%
/r/Alcoholism_Medication	***	***	***
/r/cripplingalcoholism	***	***	***
/r/cutdowndrinking	***	***	***
/r/dryalcoholics	***	***	***
/r/recoverywithoutAA	***	***	***
/r/stopdrinking	***	***	***
/r/StopDrinking	***	***	***
/r/stopdrinking/wiki/links	***	***	***
Nicotine (2 subreddits)			
**/r/StopDipping**	1086	*1176*	8,29%
**/r/stopsmoking**	*97339*	*105809*	8,70%

Cocaine, crystal meth, and other stimulants (1 subreddit)			
**/r/stopspeeding**	*7597*	10056	32,37%
Miscellaneous (1 subreddit)			
**/r/decaf**	*9675*	*12624*	30,48%
Vicodin, Percocet, heroin, and other opioid painkillers (5 subreddits)			
**/r/HeroinHeroines**	1871	2415	29,08%
**/r/Methadone**	*4930*	*7029*	42,58%
**/r/OpiatesRecovery**	22272	*24925*	11,91%
**/r/quittingkratom**	*10830*	*13456*	24,25%
**/r/suboxone**	5330	*7182*	34,75%
Miscellaneous sedatives (2 subreddits)			
**/r/quittingphenibut**	2355	*3338*	41,74%
**/r/benzorecovery**	*8907*	*12169*	36,62%
Cannabis (2 subreddits)			
**/r/leaves**	*111635*	133309	19,42%
**/r/Petioles**	*36471*	*53158*	45,75%
Recovery (12 subreddits)			
**/r/addiction**	36484	*41615*	14,06%
/r/addictionprevention	***	***	***
/r/AtheistTwelveSteppers	***	***	***
/r/easyway	***	***	***
/r/recovery	***	***	***
/r/redditorsinrecovery	***	***	***
/r/ResearchRecovery	***	***	***
/r/secularsobriety	***	***	***
/r/SMARTRecovery	***	***	***
/r/sober	***	***	***
/r/sobrietyandrecovery	***	***	***
/r/women_in_recovery	***	***	***
Therapeutic use (6 subreddits)	members 11/12/2019	members 11/05/2020	**% change**
**/r/AnAnswerToHeal**	*3081*	*3161*	2,60%
**/r/mdmatherapy**	*6856*	8276	20,71%

**/r/microdosing**	*92534*	*108277*	17,01%
**/r/PsychedelicTherapy**	5068	*6193*	22,20%
**/r/StackAdvice**	*31297*	*33569*	7,26%
**/r/Supplements**	*107951*	126614	17,29%

Harm Reduction (10 subreddits)	members 11/12/2019	members 11/05/2020	% **change**
/r/Ecinternational	***	***	***
/r/erowid	***	***	***
**/r/HPPD**	2738	3967	44,89%
/r/PillReports	***	***	***
/r/ReagentTesting	***	***	***
/r/saplings	***	***	***
/r/SSDP	***	***	***
/r/tinnitus	***	***	***
/r/TripSit	***	***	***
/r/HarmReduction	***	***	***

Cultivation (11 subreddits)	members 11/12/2019	members 11/05/2020	% **change**
**/r/Autoflowers**	*31450*	*52773*	67,80%
**/r/DMTGuide**	*10674*	*15400*	44,28%
**/r/druggardening**	*35646*	*42855*	20,22%
**/r/hempflowers**	16604	23577	42,00%
**/r/macrogrowery**	*26132*	*35498*	35,84%
**/r/MephHeads**	6382	13517	111,80%
**/r/Microgrowery**	*178048*	*207194*	16,37%
**/r/MushroomGrowers**	*40477*	*69832*	72,52%
**/r/shroomers**	*41100*	*48348*	17,64%
**/r/shrooms**	*170727*	*221673*	29,84%
**/r/Mycology**	*170056*	211101	24,14%

Discussion (47 subreddits)	members 11/12/2019	members 11/05/2020	% **change**
/r/AmsterdamEnts NL	***	***	***
/r/AskDrugNerds	***	***	***
/r/AskDrugs	***	***	***

/r/BartardStories	***	***	***
/r/bestoferowid	***	***	***
/r/Borax	***	***	***
/r/currentlytripping	***	***	***
/r/druganalytics	***	***	***
/r/druggitors	***	***	***
/r/DrugMods	***	***	***
**/r/DrugNerds**	*88195*	*98619*	11,82%
/r/drugpolicy	***	***	***
/r/drugsbooks	***	***	***
/r/DrugShowerThoughts	***	***	***
/r/drugsover30	***	***	***
/r/eldertrees	***	***	***
/r/FADQ	***	***	***
**/r/gabagoodness***	2151	3684	71,27%
/r/highdeas	***	***	***
**/r/MAOIs***	526	864	64,26%
/r/nothowdrugswork	***	***	***
**/r/ObscureDrugs***	3979	*6200*	55,82%
/r/olling	***	***	***
/r/OpiateChurch	***	***	***
/r/opiateswriters	***	***	***
/r/pharmacology	***	***	***
/r/plugging	***	***	***
/r/PsychedelicMentions	***	***	***
/r/PsychedelicMessages	***	***	***
**/r/Psychedelics**	*65956*	97612	48,00%
/r/PsychedelicStudies	***	***	***
/r/PsychoactivePoetry	***	***	***
**/r/Psychonaut**	*249642*	*282864*	13,31%
/r/PsychonautReadingClub	***	***	***
/r/psychonautreads	***	***	***
/r/psychopharmacology	***	***	***
/r/RationalPsychonaut	***	***	***
/r/RiftintotheMind	***	***	***
/r/StonerPhilosophy	***	***	***
/r/TeenageDrugs	***	***	***
/r/TheeHive	***	***	***
/r/timetolegalize	***	***	***

/r/TripReports	***	***	***
/r/TripTales	***	***	***
/r/Triptongue	***	***	***
/r/tryptonaut	***	***	***
**/r/vaporents**	*129706*	*141758*	9,29%

Misc (6 subreddits)

/r/DrugTestHelp	***	***	***
/r/DrugTests	***	***	***
/r/EntExchange	***	***	***
/r/glassheads	***	***	***
/r/mflb	***	***	***
/r/TripCaves	***	***	***

Pics & Videos (13 subreddits)

/r/currentlytripping	***	***	***
/r/DrugArt	***	***	***
/r/drugsarebeautiful	***	***	***
/r/DrugStaches	***	***	***
/r/DrugVideos	***	***	***
/r/Heavymind	***	***	***
/r/LSDselfies	***	***	***
/r/nugs	***	***	***
/r/pillhead	***	***	***
/r/psychedelicartwork	***	***	***
/r/replications	***	***	***
/r/trippy	***	***	***
/r/woahdude	***	***	***

YouTube (3 subreddits)

/r/DrugsLab	***	***	***
/r/thedrugclassroom	***	***	***
/r/PsychedSubstance	***	***	***

Memes (6 subreddits)

/r/cripplingalcoholism	***	***	***

/r/drugcirclejerk	***	***	***
/r/drugmemes	***	***	***
/r/drugscirclejerk	***	***	***
/r/drunk	***	***	***
/r/LSDcirclejerk	***	***	***
Music (9 subreddits)			
/r/DrugsMusic	***	***	***
/r/DXMMusic	***	***	***
/r/gethightothis	***	***	***
/r/lucymusic	***	***	***
/r/mdmazingMusic	***	***	***
/r/TripMusic	***	***	***
/r/WNOD	***	***	***
/r/WoahTunes	***	***	***
/r/TrippingPlaylists	***	***	***
Festivals (3 subreddits)			
/r/concertdrugs	***	***	***
/r/aves	***	***	***
/r/festivals	***	***	***
Regions/Languages (18 subreddits)			
/r/AsiaTripper	***	***	***
/r/ausents AU	***	***	***
/r/Canadients CA	***	***	***
/r/crainn IE	***	***	***
/r/DanishEnts DK	***	***	***
/r/DrogasBrasil BR banned	***	***	***
/r/drogen DE	***	***	***
/r/GermanTrees DE	***	***	***
/r/IndianEnts IN	***	***	***
/r/norwegianents NO	***	***	***
/r/NZTrees NZ	***	***	***
/r/PsychonautDE DE	***	***	***
/r/ResearchChemicalsNL NL	***	***	***
/r/rusmidler NO	***	***	***

/r/samelevel	***	***	***
/r/swedents SE	***	***	***
/r/ukdrugs banned	***	***	***
/r/uktrees GB	***	***	***

r/Drugs (main subreddit)	members 11/12/2019	members 11/05/2019	**% change**
**r/Drugs**	*619563*	*676581*	9,20%

**Other subreddits**	members 11/12/2019	members 11/05/2019	**% change**
**/r/covid19stack****	0	562	
**r/Opioid_RCs****	825	1952	136,61%

Table S2b. r/Drugs related subreddits (excluding the not analysed subreddits).

	members 11/12/2019	members 11/05/2019	**% change**
**/r/1P_LSD**	10535	*13184*	25,14%
**/r/2cb**	*17133*	*22212*	29,64%
**/r/4acodmt**	1703	2236	31,30%
**/r/5MeODMT**	1863	*2733*	46,70%
**/r/adderall**	34775	*42896*	23,35%
**/r/addiction**	36484	*41615*	14,06%
**/r/afinil**	*22947*	*23374*	1,86%
**/r/alcohol**	*69331*	*74183*	7,00%
**/r/alcoholism**	27261	*31447*	15,36%
**/r/ambien**	*65191*	95273	46,14%
**/r/aMT**	419	487	16,23%
**/r/anabolic**	1848	*2028*	9,74%
**/r/AnAnswerToHeal**	*3081*	*3161*	2,60%
**/r/Autoflowers**	*31450*	*52773*	67,80%
**/r/Ayahuasca**	*24665*	*29227*	18,50%
**/r/benzodiazepines**	*42140*	*52695*	25,05%
**/r/benzorecovery**	*8907*	*12169*	36,62%
**/r/cannabis**	*95481*	109485	14,67%
**/r/CannabisExtracts**	72511	*83064*	14,55%
**/r/Cigarettes**	*29037*	*35574*	22,51%
**/r/cocaine**	*52934*	*66890*	26,36%
**/r/covid19stack****	0	562	-

/r/decaf	9675	12624	30,48%
/r/dissociatives	3838	4742	23,55%
/r/DissonautUniverse	1357	1650	21,59%
/r/DMT	128395	157647	22,78%
/r/DMTGuide	10674	15400	44,28%
/r/DPH	4918	8417	71,15%
/r/DrugCombos	1022	1244	21,72%
/r/druggardening	35646	42855	20,22%
/r/DrugNerds	88195	98619	11,82%
/r/Drugs	619563	676581	9,20%
/r/dxm	22300	28370	27,22%
/r/fentanyl	1976	2464	24,70%
/r/gabagoodness*	2151	3684	71,27%
/r/hempflowers	16604	23577	42,00%
/r/HeroinHeroines	1871	2415	29,08%
/r/HPPD	2738	3967	44,89%
/r/Kava	9892	11589	17,16%
/r/ketamine	25801	34282	32,87%
/r/Kombucha	35487	45932	29,43%
/r/kratom	83084	92846	11,75%
/r/lean	17463	23883	36,76%
/r/leaves	111635	133309	19,42%
/r/LSA	5544	7162	29,18%
/r/LSD	327175	422806	29,23%
/r/macrogrowery	26132	35498	35,84%
/r/MAOIs*	526	864	64,26%
/r/MDMA	95371	118103	23,84%
/r/mdmatherapy	6856	8276	20,71%
/r/MemantineHCl	896	1155	28,91%
/r/MephHeads	6382	13517	111,80%
/r/mescaline	6577	14791	124,89%
/r/meth	15830	21953	38,68%
/r/Methadone	4930	7029	42,58%
/r/microdosing	92534	108277	17,01%
/r/Microgrowery	178048	207194	16,37%
/r/modafinil	11971	15633	30,59%
/r/MushroomGrowers	40477	69832	72,52%
/r/MXE	585	710	21,37%
/r/Mycology	170056	211101	24,14%

**/r/noids**	2813	*3428*	21,86%
**/r/Nootropics**	*206148*	222373	7,87%
**/r/ObscureDrugs***	3979	*6200*	55,82%
**/r/opiates**	*88356*	*98812*	11,83%
**/r/OpiatesRecovery**	22272	*24925*	11,91%
**/r/Opioid_RCs****	825	1952	136,61%
**/r/PCP**	733	1060	44,61%
**/r/Petioles**	*36471*	*53158*	45,75%
**/r/phenibut**	*23669*	25374	7,20%
**/r/PoppyTea**	*4638*	*5219*	12,53%
**/r/PsilocybinMushrooms**	21000	*29948*	42,61%
**/r/Psychedelics**	*65956*	97612	48,00%
**/r/PsychedelicTherapy**	5068	*6193*	22,20%
**/r/Psychonaut**	*249642*	*282864*	13,31%
**/r/quittingkratom**	*10830*	*13456*	24,25%
**/r/quittingphenibut**	2355	*3338*	41,74%
**/r/ResearchChemicals**	*69465*	*83554*	20,28%
**/r/Salvia**	13766	*17762*	29,03%
**/r/shroomers**	*41100*	*48348*	17,64%
**/r/shrooms**	*170727*	*221673*	29,84%
**/r/StackAdvice**	*31297*	*33569*	7,26%
**/r/steroids**	*77093*	87122	13,01%
**/r/stims**	55355	*66175*	19,55%
**/r/StopDipping**	1086	*1176*	8,29%
**/r/stopsmoking**	*97339*	*105809*	8,70%
**/r/stopspeeding**	*7597*	10056	32,37%
**/r/suboxone**	5330	*7182*	34,75%
**/r/Supplements**	*107951*	126614	17,29%
**/r/TheSaviorSubstances**	60	93	55,00%
**/r/treedibles**	*84807*	119910	41,39%
**/r/trees**	1450686	*1553229*	7,07%
**/r/vaporents**	*129706*	*141758*	9,29%

Legend

Colours

psychedelics

cognitive enhancers/PIEDS/supplements

entactogens

cannabimimetics			
dissociatives			
stimulants			
opioids			
gabaergics, gabapentinoids and various psychotropic medications			
research chemicals			
other subbreddits included among Drugs A-Z by redditors			
beverages, brews and infusions			
other psychoactive substances (cigarettes, alcohol)			

**Asterisks/slashes**
* incuded in this group by the Authors; listed in other groups by the redditors
** included in this group by the Authors; not among the r/Drugs related subreddits
*** not included in the analysis
/ impossible to obtain/no data
**Bold** = selected subreddits for qualitative and/or quantitative analysis
**Italics (numbers)** = approximate data (approximated to the nearest available data as close as possible to day 11 of each month/corrected value; approxomated excluding irrelevant posts/comments)

Table S2 summary. Member trends from December 2019 to May 2020.

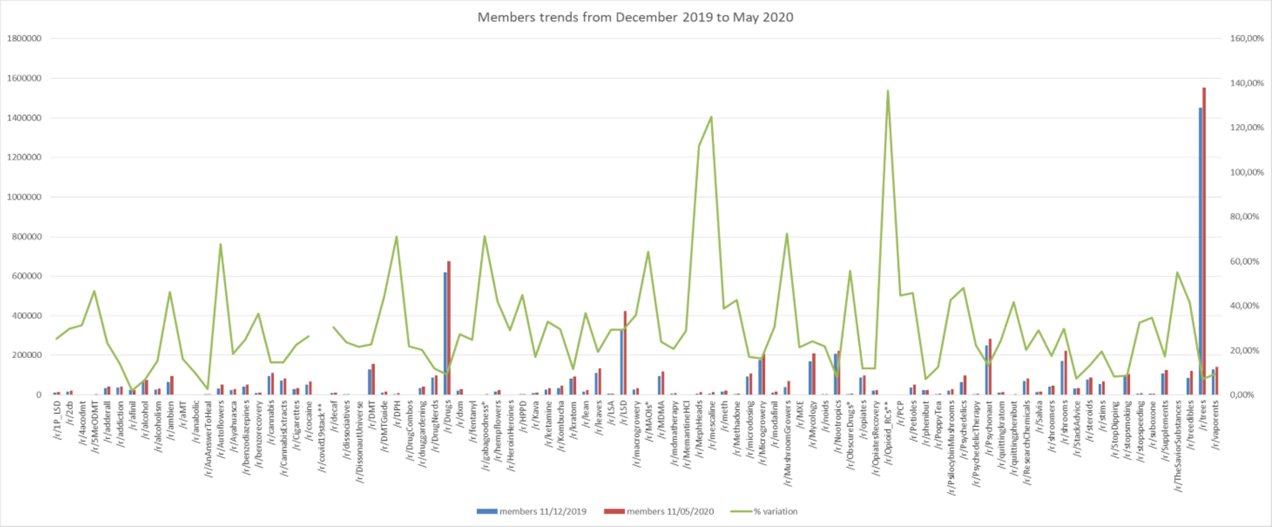

Supplement: Supplementary file 1 [file brainsci-11-00907-s001.zip › Table S4 Covid-19 impact on substance misuse psychonauts.pdf]
